# Multidimensional outlook on the pathophysiology of cervical cancer invasion and metastasis

**DOI:** 10.1007/s11010-023-04686-3

**Published:** 2023-03-11

**Authors:** Neena George, Poonam Bhandari, Padival Shruptha, Pradyumna Jayaram, Sima Chaudhari, Kapaettu Satyamoorthy

**Affiliations:** Department of Cell and Molecular Biology, Manipal School of Life Sciences, Planetarium Complex, Manipal Academy of Higher Education, Manipal, Karnataka 576104 India

**Keywords:** Cervical squamous cell carcinoma, Lymph angiogenesis, Metastasis, Genetic and epigenetic profile, Human papillomavirus

## Abstract

Cervical cancer being one of the primary causes of high mortality rates among women is an area of concern, especially with ineffective treatment strategies. Extensive studies are carried out to understand various aspects of cervical cancer initiation, development and progression; however, invasive cervical squamous cell carcinoma has poor outcomes. Moreover, the advanced stages of cervical cancer may involve lymphatic circulation with a high risk of tumor recurrence at distant metastatic sites. Dysregulation of the cervical microbiome by human papillomavirus (HPV) together with immune response modulation and the occurrence of novel mutations that trigger genomic instability causes malignant transformation at the cervix. In this review, we focus on the major risk factors as well as the functionally altered signaling pathways promoting the transformation of cervical intraepithelial neoplasia into invasive squamous cell carcinoma. We further elucidate genetic and epigenetic variations to highlight the complexity of causal factors of cervical cancer as well as the metastatic potential due to the changes in immune response, epigenetic regulation, DNA repair capacity, and cell cycle progression. Our bioinformatics analysis on metastatic and non-metastatic cervical cancer datasets identified various significantly and differentially expressed genes as well as the downregulation of potential tumor suppressor microRNA miR-28-5p. Thus, a comprehensive understanding of the genomic landscape in invasive and metastatic cervical cancer will help in stratifying the patient groups and designing potential therapeutic strategies.

## Introduction

Cervical cancer (CC) is the most common tumor of the female reproductive system and the fourth most prevalent cancer type overall [1–3]. The incidence and mortality rate of CC are the leading causes of death among women and vary among racial and ethnic groups, suggesting that genetic and epigenetic factors may determine the outcome of the disease [4, 5]. Depending on the histopathology and severity of the cervical lesion, 69% of the CC are classified as squamous cell carcinoma (SCC) and 25% as adenocarcinoma or adenosquamous cancers. The other 6% include small cell carcinoma, rhabdomyosarcoma, primary cervical lymphoma, and cervical sarcoma [5]. SCC is most likely to develop from the ectocervix and adenocarcinomas arise from the endocervix [3]. Human papillomavirus (HPV) is the key factor as the infections are detected in approximately 99.7% of the CC; however, HPV-negative aggressive CC is also identified. Persistent infections with 14 high-risk HPV genotypes are responsible for nearly all occurrences of cervical squamous cell carcinoma (CSCC) and adenocarcinoma [28]. Women infected with HPV develop precancerous phases with low-grade squamous intraepithelial lesions/CIN I (LSIL) and high-grade intraepithelial lesions or CIN II/CIN III (HSIL), and these high-grade lesions eventually lead to invasive CC over a period of time [6–8]. The role of HPV infection in tumor initiation and progression has been elegantly reviewed by Deligeoroglou et al. [29] and Steinbach and Riemer [30], and reports have been published on the mechanisms of immortalization of the host cells by HPV through signaling pathway dysregulation [11, 31–33], genomic instability [34, 35], alteration of the microbiome [36–39], and immune response modulation [40–44]. Therefore, this review only focuses on the cervical microbiome causing inflammation and discusses various events associated with CC metastasis.

Several genetic and epigenetic variations and tumor microenvironment heterogeneity contribute to malignant transformation [12, 13]. The mutation burden, copy number variations, dysregulated expression of genes, microRNAs (miRNAs), long non-coding RNAs (lncRNAs), circular RNAs (circRNAs), and DNA methylation status vary among women during different stages of CC [5, 9–11]. Further, the observed molecular landscape presents the interdependent regulatory mechanisms, which favor survival, development, progression, invasion, and metastasis. Early cancer cells develop into successive generations of cells with accumulated mutations, resulting in vigorous neoplasty. Metastasis begins with the spread of cancer cells from primary tumor sites and invading into the surrounding tissues. Furthermore, these cells can enter hematogenous or lymphogenous circulation as single cells or in clusters, represented by circulating tumor cells (CTCs). Cells then intravasate from the circulation and colonize at the secondary sites [14, 15]. In terms of biological behavior and genetics, these cells differ significantly from the primary tumor with an abnormal morphology and exponential growth [16, 17]. Several events and number of molecules are associated with metastasis. These can also be the cervicovaginal microbiome [18], immune cells in the tumor microenvironment such as macrophages and dendritic cells in association with naive T cells [19], pH of the microenvironment [20], and differential expression of oncogenic pathway proteins, such as EGFR1, Wnt/β-catenin, NF-κB, AKT, MMP3, TGF-β that may promote metastasis [21–26]. Hence, comprehensive multi-omics analysis of CC at different stages can aid in devising effective strategies for CC classification, diagnosis, and treatment. Though the early detection and treatment of CC reduces the associated risk, metastatic and invasive stages with lymphatic dissemination have poor survival statistics. Therefore, new therapeutic approaches that aid in better prognosis of CC is inevitable. In this review, initially, we discuss briefly how HPV-mediated microbiome dysbiosis supports CC progression, followed by the mechanisms underlying the pathological processes during CC cell invasion and metastasis to distant sites through lymph nodes. Furthermore, we have enumerated the somatic deregulation of key genes associated with the metastatic phenotype and their potential in preventing invasion and metastasis in CC. Hence, this study will aid in identifying potential therapeutic determinants to develop better treatment strategies and to prevent CC invasion and metastasis.

## Etiology of CC metastasis

### Dysregulation of cervical microbiome

**Fig. 1:**
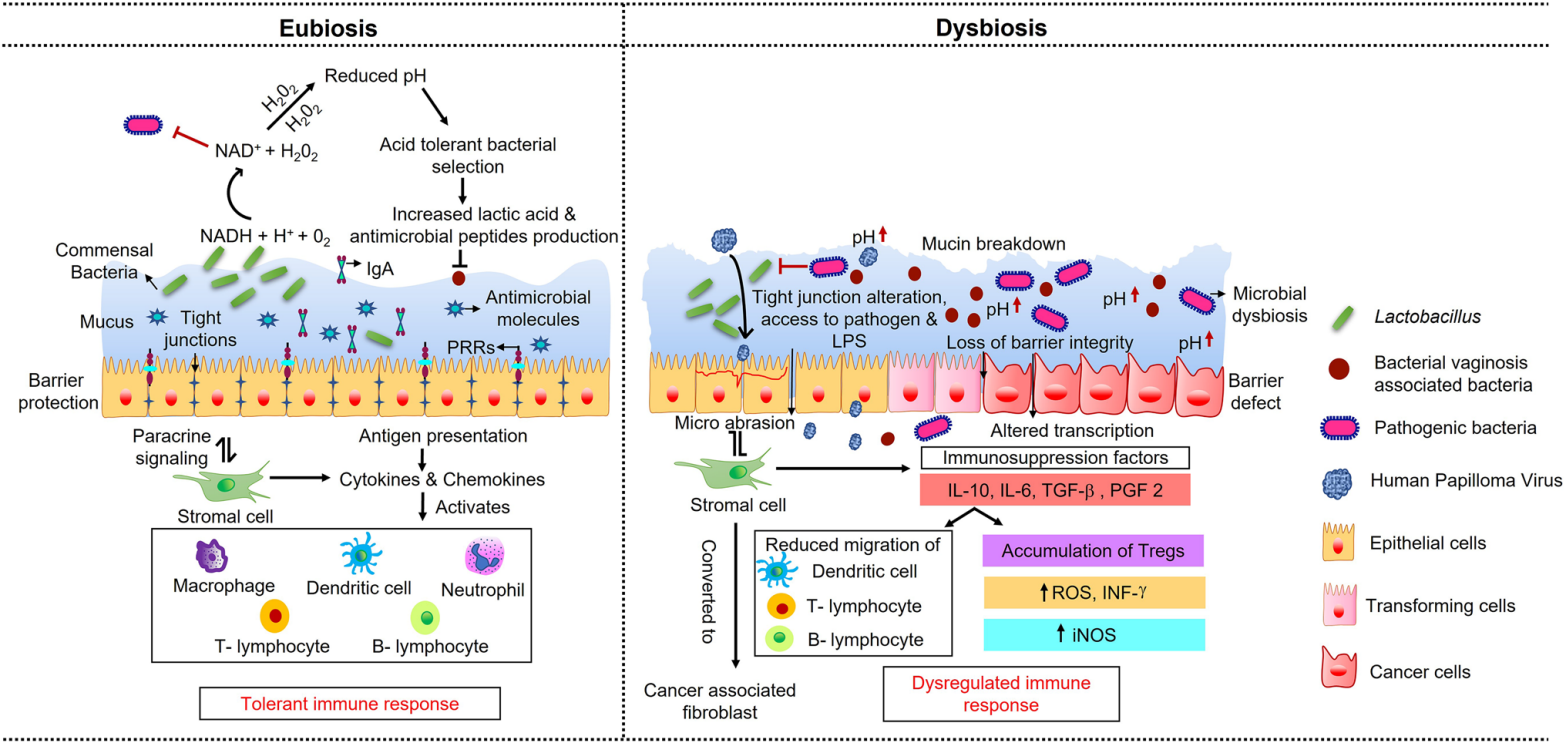
The role of cervical epithelial cells in balancing the equilibrium of the local immune system. Cervical microenvironment and the local microbial diversity alter local and systemic immune response, which play an important role in the progression of CIN toward CC. Lactic-acid-producing bacteria acidify the vaginal milieu pH to 4.6 during eubiosis, with lactic acid as the primary metabolite, thereby providing a non-inflammatory environment. The dysbiotic environment would have a lower redox potential during microbial vaginosis, a phenomenon that encourages the growth of a diverse bacterial species, resulting in the increased pH. Virulence factors produced by the diverse bacterial species undermine epithelial barrier integrity, degrade mucin, and create a pro-inflammatory milieu

It has been established that the cervicovaginal microbiome dysbiosis in concert with HPV infection contributes to CC initiation [18, 27]. The immune system at the cervical region of the uterus is governed by immunocompetent cells located along the epithelial lining of the cervix [46]. A tight regulation of innate and adaptive immunity is established in this region through a balance of the equilibrium between the ability to mount a rapid defensive immune response against invading microbial pathogens and tolerance toward commensal bacteria. During CC progression, these concerted cellular interactions are disrupted leading to a compromised immune response, resulting in low-grade chronic inflammation associated with increased susceptibility to viral and bacterial infection in various CIN stages Fig. 1 [47].

Transition of the cervical epithelium to squamous intraepithelial lesions (SIL) and CC via high risk-HPV (HR-HPV) changes the microbial diversity with an increase in the abundance of *Fusobacterium* species [48]. The immunosuppressive microenvironment initiated by the HPV infection is inhabited by *Sneathia* and other *Fusobacterium* species as it progresses to SIL. Later in the CC stage, *Fusobacterium necrophorum* colonizes abundantly and supports CC progression by modulating the cytokine profile in the tumor microenvironment [48]. Rubinstein et al. [49] reported that *F. nucleatum* is associated with colorectal carcinogenesis through FadA adhesin-mediated phosphorylation of E-cadherin, resulting in the internalization of E-cadherin together with FadA. This in turn stimulates the accumulation of β-catenin in the cytoplasm leading to the transcriptional activation of NF-κB signaling and hence promoting tumor cell proliferation. Currently, there is no conclusive evidence supporting the role of microbiome dysbiosis in CC metastasis. Therefore, further research is needed to obtain adequate knowledge about the spectrum of the cervical microbiome and its molecular determinants to envisage preventive and curative strategies for CC cell migration, invasion, and metastasis.

### Metastatic hallmarks

Metastasis is a long evolutionary multistep process that initiates the dissemination of cancer cells. Most of the cells fail to develop into a fully-grown tumor at another site, while some stay alive but fail to produce any detectable clinical manifestations and few cells initiate metastasis by evading all the immune checks. It is highly unlikely that the entire process of metastasis is regulated by a single gene. The regulation of metastasis involves activation and deactivation of various genes, and these alterations can be permanent or transient [50].

Several classes of genes have been identified, which are directly involved in metastasis including initiation of metastatic phenotype, progression, and virulence [51]. Metastasis-initiating genes help tumor cells to enter the circulation and promote cell motility, cell invasion, angiogenesis, and intravasation [52]. Further, metastasis-progression genes help in the colonization of primary tumor cells to a specific site by aiding its motility, invasion, circulatory survival, adhesion, and adaption to the new environment, as well as colonization [21]. On the other hand, metastasis-virulence genes play a role in colonization and impart aggressiveness to the tumor at the secondary site. These genes are rarely expressed at high levels in the primary tumors [53]. Recently, there is an increased focus on targeting specific pathway(s) involved in tumor growth and survival in the context of cancer treatment. Dysregulated activation of genes for the Wnt/ β-catenin pathway (*NEK-2* [54], *NUSAP1* [55], *MAGE-A3* [56]), PI3K/AKT signaling pathway (*FAM83A* [57], *FAM83H* [58], *AKIP1* [59], *KLF1* [60], *FBLN-3* [61], *TRIP4* [62], *FERMT2* [63], *SHP2* [64], *RRM2B* [65], *LHPP* [66]) and NF-κB signaling pathway (*HN1* [26], *FABP5* [67], *IFI16* [68]) as presented in Table 1, is observed in CC and may contribute to cancer cell migration and lymph node metastasis.

**Table 1:** Major signaling pathways and genes reported in CC metastasis

No.	Gene/protein	Status	Role	Pathway	Mechanism	References
1	*NEK2*	Upregulation	Oncogene	Wnt/β-catenin signaling	Lymph node metastasis	[54]
2	*NUSAP1*	Upregulation	Oncogene	Wnt/β-catenin signaling	Migration, EMT	[55]
3	*MAGE-A3*	Upregulation	Oncogene	EMT and Wnt signaling	Cell proliferation, migration and invasion	[56]
4	*HSDL2*	Upregulation	Oncogene	EMT signaling, Lipid metabolism	Cell proliferation, migration and invasion	[69]
5	*RACK1*	Upregulation	Oncogene	EMT signaling	Cell migration, invasion, lymphatic tube formation, lymphangiogenesis and lymph node metastasis	[70]
6	*CD36*	Upregulation	Oncogene	EMT signaling	Metastasis and Invasion	[72]
7	*BCAR4*	Upregulation	Oncogene	EMT signaling	Cell proliferation, migration and invasion	[73]
8	*ICAT*	Upregulation	Oncogene	EMT signaling	Cell proliferation, migration and invasion	[74]
9	*FAM83A*	Downregulation	Tumor suppressor	PI3K/AKT and TNF signaling	Inhibition of cell migration and invasion	[57]
10	*FAM83H*	Upregulation	Oncogene	PI3K/AKT signaling	Cell proliferation, colony formation, migration, and invasion	[58]
11	*AKIP1*	Upregulation	Oncogene	PI3K/AKT/IKKβ signaling	Cell proliferation, metastasis, EMT	[59]
12	*KLF1*	Upregulation	Oncogene	PI3K/AKT signaling	Cell proliferation, metastasis and invasion	[60]
13	*FBLN-3*	Upregulation	Oncogene	PI3K-AKT-mTOR signaling	Cell invasion	[61]
14	*TRIP4*	Upregulation	Oncogene	PI3K/AKT and MAPK/ERK signaling	Cell migration, invasion, Reduced radiosensitivity	[62]
15	*FERMT2*	Downregulation	Tumor suppressor	AKT/mTOR signaling	Inhibition of cell migration, Autophagy induction	[63]
16	*SHP2*	Upregulation	Oncogene	AKT signaling	Lymph node metastasis, cisplatin resistance	[64]
17	*RRM2B*	Upregulation	Oncogene	AKT signaling	Cell migration, invasion, metastasis and tumor progression	[65]
18	*LHPP*	Downregulation	Tumor suppressor	AKT signaling	Inhibition of cell proliferation, metastasis and apoptosis induction	[66]
19	*OLFM4*	Downregulation	Tumor suppressor	mTOR signaling	Inhibition of EMT, migration, and invasion	[75]
20	*HN1*	Upregulation	Oncogene	NF-κB signaling	Migration, invasion, and lymphangiogenesis	[26]
21	*FABP5*	Upregulation	Oncogene	Intracellular induced NF-κB signaling	EMT, lymphangiogenesis, Lymph node metastasis	[67]
22	*IFI16*	Upregulation	Oncogene	STING-TBK1- NF-κB signaling	Cell migration and invasion	[68]
23	*CXCR7*	Upregulation	Oncogene	CXCL12/CXCR7 signaling	Cell proliferation and invasion	[76]
24	*PBK*	Upregulation	Oncogene	ERK/c-Myc signaling	Cell invasion and migration	[77]
25	*RAP2B*	Upregulation	Oncogene	ERK1/2 signaling	Cell proliferation, migration, invasion and metastasis	[78]
26	*SND1*	Upregulation	Oncogene	SND1-induced FOXA2 ubiquitination	Cell migration, invasion and EMT, Lung metastasis	[79]
27	*SEMA4C*	Upregulation	Oncogene	TGF-β1-induced p38 MAPK activation	EMT induction, invasion and metastasis	[80]
28	*SIRT3*	Upregulation	Oncogene	Fatty acid metabolism	Cell migration and invasion	[81]
29	*NSD2*	Upregulation	Oncogene	TGF-β1/ TGF-βRI/ SMADs signaling	Cancer progression and metastasis	[82]
30	*ZFP42 (REX1)*	Upregulation	Oncogene	JAK2/STAT3-signaling	Metastasis, EMT induction	[83]
31	*CSN6*	Upregulation	Oncogene	Autophagic degradation of CTSL	Cell migration and invasion	[84]
32	*S100A7*	Upregulation	Oncogene	RAGE mediated ERK signaling	Migration, invasion, Metastasis and EMT induction	[85]
33	*TRIO*	Upregulation	Oncogene	RhoA/ROCK signaling	Cell migration and invasion	[86]
34	*SH3BP1*	Upregulation	Oncogene	SH3BP1/Rac1/Wave2 signaling	Invasion, migration, and chemoresistance	[87]
35	*YB-1*	Upregulation	Oncogene	YB-1/SNAIL/epithelial-mesenchymal transition axis	Cell invasion, EMT induction	[88]
36	*EZR*	Upregulation	Oncogene	–	Cell migration and invasion	[89]
37	*EHMT2*	Upregulation	Oncogene	–	Cell proliferation, adhesion and invasion, Apoptosis induction	[90]
38	*ZAC1*	Upregulation	Oncogene	–	EMT induction, Migration	[91]

### Lymph angiogenesis: progression of cervical cancer metastasis

Microvascularization is an early regulation, which is inevitable during the gradual progression of CSCC. Numerous signaling cascades, which can be attributed to the dysregulation of various genes, contribute majorly for the development of angiogenesis and lymphangiogenesis, which is indispensable for the progression of CIN toward invasive CSCC. Pathological changes associated with tissue inflammation or tumor progression are involved in the aberrant angiogenic and lymphangiogenic development, which is a prerequisite for the dissemination of the tumor to distinct sites [92, 93]. Hematogenous and lymphogenous spread of tumor cells are the two major routes well studied for cancer dissemination and are associated with poor prognosis. Although blood and lymph vessels share same origin, they have differences in their structure and function. Lymphatic vessels are derived from the vascular endothelium, whose formation is regulated by Prospero homeobox protein 1 (PROX1), a lymphatic specific transcription factor [93, 94]. Association of PROX1 with COUP-TFII upregulates the vascular growth factor receptor-3 (VGFR-3) signaling by vascular endothelial growth factor-C (VEGF-C) and allows the migration of cardinal vein lymphatic progenitor cells to form primary lymphatic plexus at the adjacent mesenchyme. This migration of the progenitor cells is balanced by the collagen and calcium-binding EGF domain1 protein. Lymph angiogenesis is then initiated via VEGF-C signaling [93]. On the other hand, angiogenesis is regulated by VEGF-A through VEGFR-1 and VEGFR-2 receptor signaling [95]. The lymphatic vessels have approximately threefold wider lumen than the blood capillaries, and the tumor cells prefer to disseminate through the lymphatic system due to the high levels of hyaluronic acid, which gives protection from the blood serum toxicity and provides a safe path for the migrating cells. The low flow rate of the lymph causes minimum shear stress to the cells, which makes it an energy-efficient mechanism for the evading cells. The lymph endothelial cells with a leaky arrangement on the surface of the lymphatic capillaries and lacking basement membrane will in turn support the easy migration of the tumor cells to their target sites [94]. Metastasized tumor cells will have additional traits such as mutations and genetic heterogeneity to overcome the lymphatic barrier. Analysis of the tumor driver genes and their associated mutations are highly correlated with cell survival, cell fate, and maintenance of genome [96]. Genetic heterogeneity due to the unstable genome of the subpopulations of cells can expand and cope in adverse environment and affect tumor evolution. Genetically modified subpopulations can entirely suppress the growth of primary clones imparting resistance to therapy and immune checks [97]. In most solid tumors, metastasis through the lymphatic system is observed in the early stage where the infiltrated tumor cells can either migrate to different sites or remain dormant in the lymph vessel.

### Molecular determinants of lymphatic dissemination in CSCC

The role of lymph node in CC is recognized and is included in the examination of lymph node metastasis (LNM) for staging the CC by the International Federation of Gynaecology and Obstetrics (FIGO) classification in 2018. Occurrence of LNM in the pelvic region reflect stage IIIC1 while that in para-aortic region reflect stage IIIC2 [98, 99]. CC is mainly known to metastasize utilizing three main routes—direct invasion into the neighboring tissue, hematogenous dissemination, and lymphatic dissemination [5]. Pelvic and para-aortic lymph nodes are the first sites for tumor draining and nodal metastasis in CC [100, 101]. Further, high lymphatic vessel density (LVD) with elevated levels of lymphangiogenic factors such as VEGF-C and VEGF-D are identified in pre-invasive neoplasia (CIN3) [5, 102–104]. VEGF, an angiogenic factor with well-established prognostic value in gynecologic cancers [105], is overexpressed in early stage of CSCC and is positively correlated with microvessel density (MD) at the early stage of CSCC. This suggests the role of VEGF as a potential marker for developing benign tumor to an invasive state. VEGF is also linked to the incidence of LNM of CSCC [106, 107]. Studies have also shown that hypoxia-induced transcription factor (HIF-2α) with its increased expression in association with VEGF reduces the patient survival rate in CSCC. This correlation between HIF-2α and VEGF was clearly observed in tissue samples with high FIGO stages of LNM [108]. VEGF activation can also be mediated by the guanine nucleotide exchange factor (GEF) NET1 in CSCC without influencing MMP2 and MMP9 expression. NET1-mediated activation of RHOA, FAK, JNK, NF-κB and Wnt signaling was observed during the initiation and progression of various cancers. Among the risk factors, LNM indicates poor prognosis at early or late stages of CSCC [109] and higher the risk of recurrence. Similarly, along with VEGF expression, metabolic parameters such as total lesion glycolysis (TLG) are significantly higher during lymphatic metastasis of CSCC. Therefore, combined evaluation of the TLG and VEGF may help in predicting the LNM [110].

**Fig. 2:**
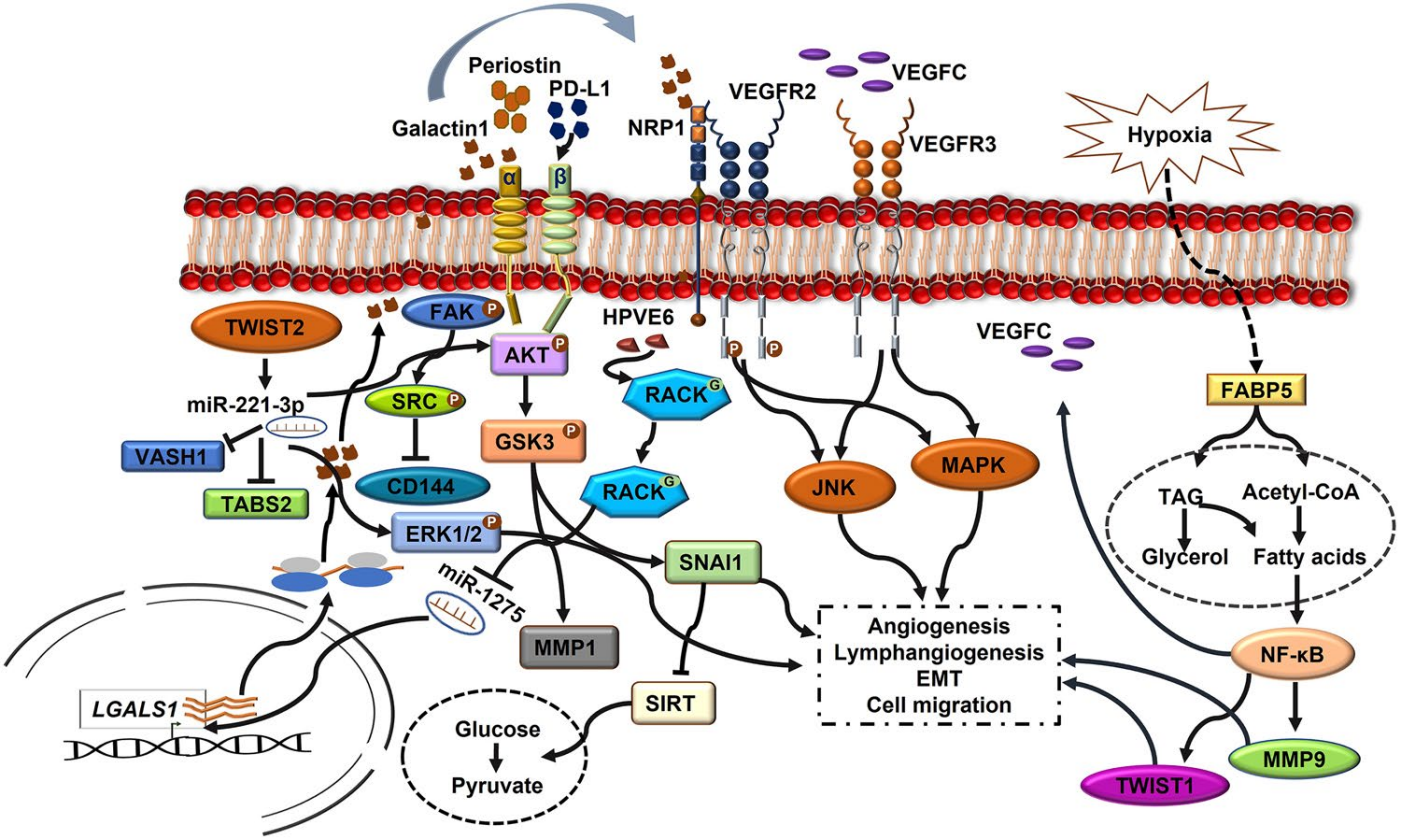
Illustration showing the molecular signaling mechanisms by which CSCC undergoes epithelial–mesenchymal transition, lymph angiogenesis, and metastasis via the lymphatic system

The receptor for activated C kinase 1 (RACK1), a scaffold protein overexpression in CC, showed increased ability for lymph node metastasis. HPV16/18 proteins E6 and E7 have shown significant association with the upregulation of RACK1 in CC with an increased expression of N-cadherin and SNAIL and downregulation of epithelial markers such as E-cadherin and ZO-1 [70]. E6 stabilizes RACK1 through O-GlcNAcylation at Ser122 and promotes tumor invasion and metastasis by inhibiting miR1275, which inhibits *LGALS1* gene that encodes galactin1. Therefore, inhibition of miR-1275 increases galactin1 expression, which in turn activates MEK/ERK, FAK and AKT signaling involving integrin-β1 and promotes LNM in CSCC [70, 71]. Previous evidence has shown that the galectin1 activates NRP1 signaling to phosphorylate VGFR2 during endothelial vascular cell migration and angiogenesis [111]. Additionally, the activation of miR-221-2p is also positively correlated with LNM through the downregulation of THBS2 protein [112]. The enriched expression of miR-221-3p found in CSCC-secreted exosomes inhibits the expression of *VASH1* by lymphatic endothelial cells and induces lymph angiogenesis and LNM. The increased density of peritumoral lymphatic vessel density (PLVD) than MVD indicates that CSCC prefers lymphatic mode of metastasis than hematogenous dissemination. miR-221-3p also increased the phosphorylation of AKT and ERK1/2 proteins independent of VEGF-C, demonstrating that miR-221-3p—*VASH1* axis share common pathway with VEGF-C in lymphatic vessel sprouting and metastasis [113]. This indicates that miR-221-3p is involved in context-dependent activation of microvessel development. The molecular changes in the tissue biopsy show an upregulation in EMT-inducing transcription factors TWIST1 and SNAIL in association with metastasis and lymphovascular space invasion (LVSI) Fig. 2 [114]. Further, studies have correlated the expression of lncRNA with cellular invasion in CC, which explains their potential role as a biomarker for cancer metastasis. Mining the lncRNA profile of human cancer revealed 234 lncRNAs associated with pelvic lymph node metastasis (PLNM). Among these lncRNAs, MIR100HG and AC024560.2 are highly associated with the deregulation of gap junction proteins. The aberrant expression of gap junction proteins assists LNM in ductal breast cancer, oral SCC and ovarian adenocarcinoma. Validating the expression of these lncRNAs in CC tissues may provide promising evidence for their diagnostic potential at an early stage [121]. Another study by Shang et al. [127] demonstrated the role of lncRNA regulation in fatty acid metabolism and tumor metastasis in cancer stem cells (CSCs). Reprogramming of fatty acid metabolism by lncRNAs mediated through FABP5 in CC activates VEGF-C and promotes LNM.

### Tumor microenvironment in lymphatic dissemination of CSCC

Tumor-associated macrophages (TAMs), matrix metalloproteinases, interleukin-2 (IL-2) and TGF-β present in the microenvironment are the key factors involved in lymphatic dissemination [115–119]. Oany et al. [120] reported the upregulation of immune and inflammatory response genes in CC. High percentage of protein tyrosine phosphatase receptor-type C^+^ (PTPRC^+^) cells in cervical tumor are associated with enhanced tumor-infiltration by T-BET^+^ cells and FOXP3^+^ cells [120]. In addition, the tumor microenvironment participates in tumor dissemination by increasing the permeability of the endothelial barrier of the lymphatic vessels [106]. Recently, research has advanced in elucidating the role of cancer associated fibroblasts (CAFs) in tumor progression. Reconstitution of CAFs enables the non-metastatic tumor to undergo proximal LNM in CC cells co-transplanted in athymic mice [122]. Wei et al. [124] demonstrated that the metastasis of CSCC to the lymph node is highly influenced by the subpopulations of CAFs in the tumor microenvironment. They have identified a specific subset of CAFs with elevated periostin expression, which activates integrin αvβ3 and αvβ5 signaling and triggers the phosphorylation of FAK (Tyr397) and SRC (Tyr416) that results in the direct degradation of VE-cadherin. These may breach the endothelial barrier and establish LNM in CSCC. Therefore, identifying protumorigenic CAFs and targeting the FAK-SRC axis could form an efficient target for the prevention of tumor metastasis [124]. Another important factor associated with lymph metastasis is the extracellular matrix (ECM) remodeling. ECM component includes network of collagen, fibronectin, laminin, elastin and proteoglycans, which provide structural and mechanical stability to the tissue and regulate the availability of cytokines and growth factors in the microenvironment. In CSCC, HPV E6/E7 modulates carcinogenic process by regulating the ECM protein CTHRC1 and upregulating its expression in CC tissue. E6/E7 expression activates POU2F1 by inhibiting p53, which in turn activates CTHRC1-mediated Wnt/PCP signaling pathway and promotes cancer cell migration and invasion [125]. Studies have also showed that overexpression of MMP-2 (gelatinase A) and MT1-MMP (collagenase) by HPV16 E6/E7 facilitates ECM degradation and cancer invasiveness [126].

### Immune evasion of CSCC in LNM

Primary lesions disseminate into the lymphatic drainage through sentinel lymph node (SLN) [128]. The migratory antigen-presenting macrophages or dendritic cells activate CD8^+^T cells in lymph node tumor-draining site, which provides first line of defense [128–130]. However, developing tumor cells target tumor-draining lymph nodes (TDLNs) to create a pre-metastatic niche with suppressed immune response, harboring dysfunctional anti-tumor T cells [131]. The increase in the level CD1a^+^ dendritic cells at the TDLN provides evidence that dendritic cell maturation is inhibited at the lymph node by tumor-derived growth factors, such as prostaglandin-E2 (PGE2), TGF-β, IL-6, and VEGF [128, 132–136]. Further, blood vessel remodeling, lymphangiogenesis, and increased chemokine and cytokine secretion alter the immune cell composition and give rise to a ‘tumor-supportive’ microenvironment [131]. Moreover, high Treg counts along with increased cytokine release and a consistent immune suppressive microenvironment were observed with high levels of TNF-α, IL-6, IL-10 and reduced IFN-γ expression [128]. Metastasis was preceded by low CD8^+^T cell/FoxP3^+^Treg ratios, creating pre-metastatic niches in the tumor-draining lymphatic basin [137]. The recruitment [138] and polarization of CD14^+^ monocytes into the suppressive PD-L1^+^M2-like macrophages (co-expressing CD14 and/or CD163) by primary tumor cells (possibly via the secretion of CCL2) prevent the antigen presentation and immune response in the lymphatic niche [139]. These M2-macrophage-like cells are not only incapable of generating proper CD8^+^ T cell responses but favor Treg expansion, and facilitate the production of pro-angiogenic and pro-tumor-invasive factors [128, 140, 141]. Elevated rates of Tregs and CD4/CD8 T cell ratios were observed prior to metastatic involvement in cervical TDLN [142]. As the metastasis progresses in TDLN, myeloid regulatory cells and memory T cells accumulate resulting in the release of exosomes carrying immune modulatory elements and soluble mediators, leading to “tumor-supportive” microenvironment [131, 142, 143]. The accumulation of Tregs inhibits lymph node-resident conventional dendritic cell (LNR-cDC) activation causing the conversion of Ag-specific naive T cells into Tregs in TDLN [143, 144].

Transcriptomic analysis of CC data from The Cancer Genome Atlas (TCGA) by Yang et al. [145] suggested the association of LNM with upregulation of immune biomarkers. In high-risk group, LNM was associated with *FABP4* and *CXCL2* upregulation, while *TEKT2* and *LPIN2* were downregulated [145]. Upregulation of FABP4 promotes EMT by activating AKT pathway [146], whereas CXCL2 promotes tumor growth and angiogenesis via NF-κB signaling [147]. Expression of CXCL2 is positively correlated to the neutrophil activation and poor prognosis. Additionally, the expression of PD-L1 was upregulated in high-risk group and promotes CC metastasis to the lymph node [69, 148]. PD-L1 is characterized for its role in masking the tumor cells by binding to the PD-1 receptor expressed on the T cells and inhibiting immune response, thereby creating an immunosuppressive microenvironment [149]. PD-1 phosphorylates and activates PD-L1-integrinβ4 (ITGB4) signaling pathway followed by the activation of AKT-GSK3 pathway. This results in the upregulation of SNAI1 and MMP1 and decrease in the cytokine production [150]. The PD-L1-integrin axis also increases the glucose metabolism and tumor cell proliferation through SNAI1-mediated downregulation of SIRT3 in CC [148], suggesting that the reprograming of Warburg effect is essential for the tumor cell dissemination.

### Migration and colonization of CSCC to distinct organs

According to the SEER database analysis from 2010 to 2015, there are four major sites for CC metastasis. Lungs are the usual site of metastasis and colonization with a rate of 56% followed by liver with 16%, bone with 23%, and brain with 2% [151]. Cox multivariate analysis of the patient data obtained from SEER database confirm that increasing age, non-squamous type, advanced stage, metastasis through pelvic lymph node and poor differentiation are risk factors for lung metastasis [152]. It was reported that the pulmonary metastasis occurs heterogeneously and in most of the cases the patients are asymptomatic [152, 153]. Studies on the metastatic niches of CC in large population showed lungs as the most usual site for colonization. Major challenge is to differentiate lung SCC from metastasized CSCC. It is reported that HPV modulates p16 upregulation and suppresses pRB-E2F signaling by inhibiting CDK4 cyclin-dependent kinase. In CSCC, p16 expression is relatively higher than pulmonary SCC as it is upregulated by HPV infection [153, 154]. Therefore, along with p16 expression, the presence of HPV DNA is used for differentiating the metastasized SCC from the lung SCC [154]. When the malignant tumor cells enter metastasis, the surviving cells may infiltrate to the distant organs. These infiltrated cells relapse at their new niche, eventually proliferate to form tumor, and distort the function of the host organ. Colonization of the host organ is the slowest step of tumor dissemination, and there is no adequate reference for the mechanism by which CSCC infiltrate to its distant organs. But recent advances in the technologies to access the detailed insight of the CTCs help to conceptualize the triggers for organ infiltration [155]. CTCs modify the chemokine gradient for tumor invasion and colonization. This invasive front is comprised of myeloid progenitor cells, TAMs, CAFs and newly formed blood vessels [156] along with Notch, Wnt, TGF-β and cytokines signaling [157].

### Cytokine–chemokine gradient driving tumor invasion to host niche

Disseminating tumor cells (DTCs) migrate as single cells or as a group. The success in the establishment of tumor metastasis depends on the ability of CTCs to overcome various hindrance in the hematogenous or lymphogenous pathways. Emerging studies have shown that collective migration of the metastatic cells has higher invading ability and successfully establishes tumor migration to distinct organs. They move in clusters possessing strong adhesion along with the tumor microenvironment, which may provide resistance toward clinical interventions [155, 158, 159]. Recently, Pein et al. [159] demonstrated the role of metastasis-associated fibroblasts (MAFs) in migration and colonization of breast cancer cells to the lungs by modulating the expression of chemokines. For the evolution of a supportive niche in the lungs, migrating breast cancer cells secrete interleukins IL-1α/β. Additionally, they also observed a subset of lung fibroblasts that expresses CXCR3, which binds to CXCL9/10. The secreted interleukins induce chemokines CXCL9/10 in the MAFs via NF-κB signaling. CXCL9/10 fuel the proliferation and metastatic outgrowth in lungs by activating the MAFs and support the colonization of CTCs in the lungs [159]. Chemotactic signals modulate the interaction of the cancer cells within the cluster and its migration into host organ. Another study on the role of CXCL17 in the lung metastasis of the breast tumor cells provides additional evidence to substantiate the chemokine facilitation of tumor dissemination. CXCL17 induces accumulation of the CD11b^+^Gr-1^+^ myeloid-derived suppressor cells (MDSCs) in the lungs and promote tumor angiogenesis and extravasation of cancer cells to the lungs through the overexpression of PDGF-BB [160]. These explain the engagement of chemotactic signals in the colonization of primary tumors and could be the possible mechanism supporting CC metastasis and colonization in pulmonary tissue. Another study demonstrated that the overexpression of NRAS in the CTCs promote lung colonization by IL-8 mediated the expression of CXCL5 and pro-platelet basic protein (PPBP). These factors may target the CXCR1 receptor of pulmonary blood vessel and facilitate the adhesion of the tumor cells to the lung vasculature as well as the recruitment and homing of CXCR2 receptor-expressing myeloid cells to the microenvironment. Thus, chemokine secretion-mediated activation of CXCR1/2 receptor at the metastatic site helps the CTCs to form pre-metastatic niche in the lungs [161].

The incidence of bone metastasis in CC is relatively rare and increases with the advanced stage. Previously, Matsuyama et al. [162] reported the association of bone metastasis of CC with its clinical stages among stage I (4%), stage II (6.6%), stage III (8%) and stage IV (22.9%) [162] of bone metastasis with high frequency to the lumbar spine and to the pelvic bones [162, 163]. A case report of 70-year-old women with FIGO stage IIIA also showed metastasis within fibula, calcaneum and right tibia [164]. The ECM composition of the bone favors the cancer cells to colonize and proliferate in the bone as it tightly controls the bone remodeling. The major organic component of bone ECM is collagen1, which is involved in providing bone strength and stiffness [165] and also supports survival, proliferation and differentiation [166]. The accumulation of collagen type I at the pre-metastatic niche of the host organ distorts the alignment of ECM and supports the colonization of CTCs [165, 167]. Like the lung colonization of breast cancer cells, chemotactic paracrine signaling of CXCL5 induces bone metastasis via activation of CXCR2 receptor on the bone marrow [168]. Primarily, chemokine gradient produced by the CTCs shapes the tumor microenvironment at the host pre-metastatic niche, promoting angiogenesis and recruiting antitumoral leukocytes, TAMs and MDSCs to support the metastatic invasion of host site. The studies have shown that the secretion of endothelin-1 by the circulating breast cancer cells modulates TGF-β production in osteoblast leading to bone metastasis [169]. The metastasis of cervical cancer directly to the brain is a rare occurrence [170, 172]; instead, spreading of the tumor cells to the lungs as it is the most common site and subsequently to the brain is observed [170]. Another possible pathway for dissemination is from the pelvic veins to the vertebral venous plexus and then to the brain parenchyma via the venous sinus of the brain [171].

Liver metastasis was found to be significantly correlated to brain, lung and bone metastasis, as liver has rich blood supply from portal as well as arterial venous system making cancer cells to spread easily [173]. The incidence of liver metastasis is equally low, accounting for only 1.2–2.2 percent of all cases and only 5% of the case developed hepatic metastasis alone without any extrahepatic comorbidity [174]. The liver metastasis occurs later in the progression of primary CC; thus, early surgical excision of primary lesions can successfully prevent hepatic metastasis [175]. Current understanding on the mechanism of distinct organ metastasis by CC cells is very limited. Further studies are warranted to establish the characteristic features associated with CTCs of CSCC and its colonization and will help to unravel the mechanisms promoting CC cell migration and colonization.

### Genetic and epigenetic features in cervical cancer metastasis

Initiation and development of CC is a complex process [8]. Although HPV infection is the critical determinant for the risk of developing CC, not all women infected with HPV develop CC. Host genetic variants and environmental factors also add to the risk towards the susceptibility to CC [176]. Further, the genomic and epigenomic profiling of CC at different stages along with the gene expression analysis supports the role of these events in CC initiation, progression, invasion and metastasis [9, 10]. Hence, comprehensive genomic and epigenomic land scape of CC can help in stratification of affected individuals and may facilitate effective treatment strategies [10].

### Genetic predisposition in cervical cancer

A large number of genetic variations are observed in CC, and the burden of variations increases with high grade and severity of the disease [9]. The genetic variations observed in cancer may be the germline that predispose individual for risk of CC [179]. Previous candidate gene-based studies reported polymorphisms in genes associated with immune response [human leukocyte antigen (HLA), tumor necrosis factor-α (*TNF-α*), interferon-γ (*IFNG*), cytotoxic T-lymphocyte antigen-4 (*CTLA- 4*), interleukins (*IL-1β, IL-12β, IL-10*)]; pathogen gene response [Toll-like receptor (*TLR2, TLR3, TLR4, and TLR9*]; DNA repair or cell cycle [*ATM, BRIP1, CDKN1A, CDKN2A, FANCA, FANCC, FANCL, XRCC1, XRCC3*]; apoptosis (*FAS, FASL, CASP8, TP53, MDM2*); antigen-processing gene (*LMP, TAP, ERAP*); xenobiotic metabolism, and other processes are associated with CC susceptibility [177]. With the advent of high-throughput technologies, genome-wide association studies (GWAS) have been conducted across the population of different ethnicity, and multiple CC susceptibility loci have been identified that overlap among the different populations. The most significant loci are 6p21.3 [*HLA* locus]; 2q13 (*PAX8*), 5p15.33 (*TERT-CLPTM1L*), and 17q12 (*GSDMB*). Polymorphisms in *ARRDC3, INS-IGF2, SOX9, TTC34, ACACB* have also been associated with a risk of developing CC; however, this remains to be validated [176, 178]. Several of these polymorphic genes are potential candidates for immune evasion in distant sites and hence have the ability to promote metastasis.

Although mutations are prevalent in CC, only a few can act as driver mutations, which are expected to initiate and promote growth, and these are common mutations identified in cancer invasion and metastasis [9]. Additionally, HPV infection can result in increased mutational spectrum [41]. Furthermore, when metastasized to unrelated lineage microenvironment, the tumor cells can acquire new mutations [9], which are genotypically favorable for malignant cells [180]. Genomic profiling of CC patients reported frequent mutation in *EP300, MUC4, MUC16, SYNE1, KMT2C, PIK3CA, FLG, KMT2D, DST, MAPK1,* and *TTN* [10]. Also, the mutation signatures depict DNA mismatch repair deficiency (COSMIC Signature 6), APOBEC cytidine deaminase (COSMIC Signature 2), and spontaneous deamination of 5-methyl cytosine (COSMIC Signature 1) pattern [10]. Integrated molecular profiling of primary and recurrent/metastatic CC from same individual reported elevated mutation burden and copy number alteration. Mutations in epigenetic regulators such as *NSD1, ARID1A, CTCF, ARID1B, KMT2C, SETBP1, PBRM1,* and *KMT2D* were specific to recurrent/metastatic CC [9]. APOBEC-related mutation signature along with reduced expression of *APOBEC3A* was also observed for recurrent/metastatic CC. Also, in some cases of CC, nuclear-encoded sigma factor 6 (SIG6) mutation is associated with defect in mismatch DNA repair [9].

### Epigenetic alterations in cervical cancer

Non-coding regions within the genome have a major impact on the progression of cancer. Deregulated expression of small miRNAs as well as lncRNAs and circRNAs has been associated with distinct stages of CIN and development of CC [11]. Specifically, miR-27a, miR-21, miR-34, miR-196a, and miR-34a are highly expressed in SCC [182, 183]. The dysregulation of miRNA expression, its role in CC pathogenesis, invasion, and metastasis are well reviewed earlier [184–187]. Zhang et al. [1] reported that the downregulation of miR-320 upregulates *MCL-1* leading to the progression of CC by evading apoptosis signaling [1]. Expression of miR-320a is also correlated with the downregulation of *FOXM1*. However, circCLK3 sponges miR-320a, thus resulting in increased *FOXM1* expression and significant promotion of cell metastasis in vitro as well as in vivo [194]. miR-320a is also correlated with LNM [195]. Similarly, circSLC26A4 and circGSE1 promote CC progression through miR-1287-5p/HOXA7 axis and miR-138-5p/Vimentin, respectively [196, 197]. CircNRIP1 sponges miR-629-3p and promotes invasion and migration by regulation of PTP4A1/ERK1/2 pathway [198]. The HOX Antisense Intergenic RNA (HOTAIR), a lncRNA, inhibits *p21* expression, regulates expression of *MMP-9*, *VEGF* and genes related to EMT, all of which are essential for migration and invasion of CC [5, 188]. Recently, eight lncRNA signatures, including LINC01990, RUSC1-AS1, LINC01411, H19, LINC02099, LINC00452, C1QTNF1-AS1, and ADPGK-AS1, involved in poor prognosis have been identified through an integrated multi-omics approach [189]. Additionally, constitutive expression of HPV16-E7 protein enhances the expression of lncRNAs such as CCEPR [191], MALAT1 [192], and TMPOP2 [193], thereby promoting CC progression and potentially aiding in metastasis.

Our bioinformatic analysis of TCGA data of CC revealed reduced expression of miR-28-5p in CC patient (unpublished data). miR-28-5p is located at 3q27.3, which is frequently gained in CC. The significant downregulation in CC could be due to circRNA ArfGAP with FG repeats 1 (circAGFG1), which binds to miR-28-5p and targets *HIF-1α*, thus promoting proliferation, invasion, and migration as well as suppression of apoptosis by escalating glycolysis through circAGFG1/miR-28-5p/HIF-1α axis as observed in non-small-cell lung cancer (NSCLC) [199]. Furthermore, lncRNA CDKN2B antisense RNA 1 (CDKN2B AS1) which is known to be upregulated in colorectal cancer has been reported to bind miR-28-5p in order to regulate proliferation as well as apoptosis inhibition [200]. Similar mechanisms of regulation may be evident in CC hence downregulation of miR-28-5p could promote CC progression and invasion. This evidence also suggests the intricate and interrelated regulatory pathways that exist for the suppression or the progression of CC [38, 45, 181, 201, 202].

### Metastasis-regulated differentially expressed genes

GSE26511 dataset from TCGA including 39 samples (20 CC-lymph node-negative samples and 19 CC-lymph node-positive samples) was analyzed by Ge et al. [204]. Approximately 1,263 genes were differentially expressed, and these genes were associated with signaling pathways, cell cycle processes, immune response, regulation of immune system processes, inflammatory response, and cell activation [204]. In PLNM, p120-associated non-canonical β-catenin pathway and TGF-β were important [204]. Further investigation of cancer-associated pathways revealed dysregulation of five pathways (NFAT, TGF-β, ALK, PAR1, and BAD) in CC-lymph node-negative samples, while CC-lymph node-positive samples showed deregulated glycosphingolipid biosynthesis neolacto-series and β-catenin pathways [204].

Dataset analysis of 116 non-metastatic and 10 metastatic samples from “TCGA” indicated differential expression of transcription factor *NR5A2* in metastatic tissue compared to non-metastatic tissue of CC [3]. Earlier, *NR5A2* had been identified to contribute in developing CC during GWAS [10, 205]. NR5A2 plays an important role in maintaining pluripotency in embryonic stem cells (ESCs) [206] and reprogramming of somatic cells into induced pluripotent stem cells (iPSCs) [207]. The genetic heterogeneity among the CC, recurrence and radio/chemotherapy resistance, tumor invasion and metastasis are attributed to the presence of CSCs [208, 209]. CSCs have self-renewal and multi-lineage differentiation abilities [210]. Significant and positive correlation was observed between NR5A2 and vimentin [3]. Vimentin is the biomarker of EMT, which is the first step for invasiveness and metastasis [211]. EMT is characterized by the reduced expression of E-cadherin (epithelial marker) and increased expression of N-cadherin and vimentin (mesenchymal marker) [3, 212]. Thus, NR5A2 positively regulates vimentin and activates EMT signaling pathway in metastatic CSCC [3]. Transcription factors associated with EMT, such as SNAI (SNAI1 and SNAI2), ZEB (ZEB1 and ZEB2), and TWIST (TWIST1 and TWIST2), can suppress expression of E-cadherin and regulate the EMT through different pathways [213]. Matrix metalloproteinase-1 (MMP1) degrades ECM during both physiologically normal and disease processes. In CC, it is upregulated and aids in cancer cell invasion, migration and proliferation via EMT. MMP1 is closely linked with LNM [203, 214]. Differential gene expression analysis of primary and recurrent/metastatic CC from the same individual suggested upregulation of activated anti-tumor immunity gene in primary CC and genes involved in EMT and angiogenesis in recurrent/metastatic CC. While *CXCL9, SPEG, MUC21*, and *APOBEC3A* were downregulated, *POSTN* was upregulated in metastatic CC compared to primary tumor [9, 203].

**Fig. 3:**
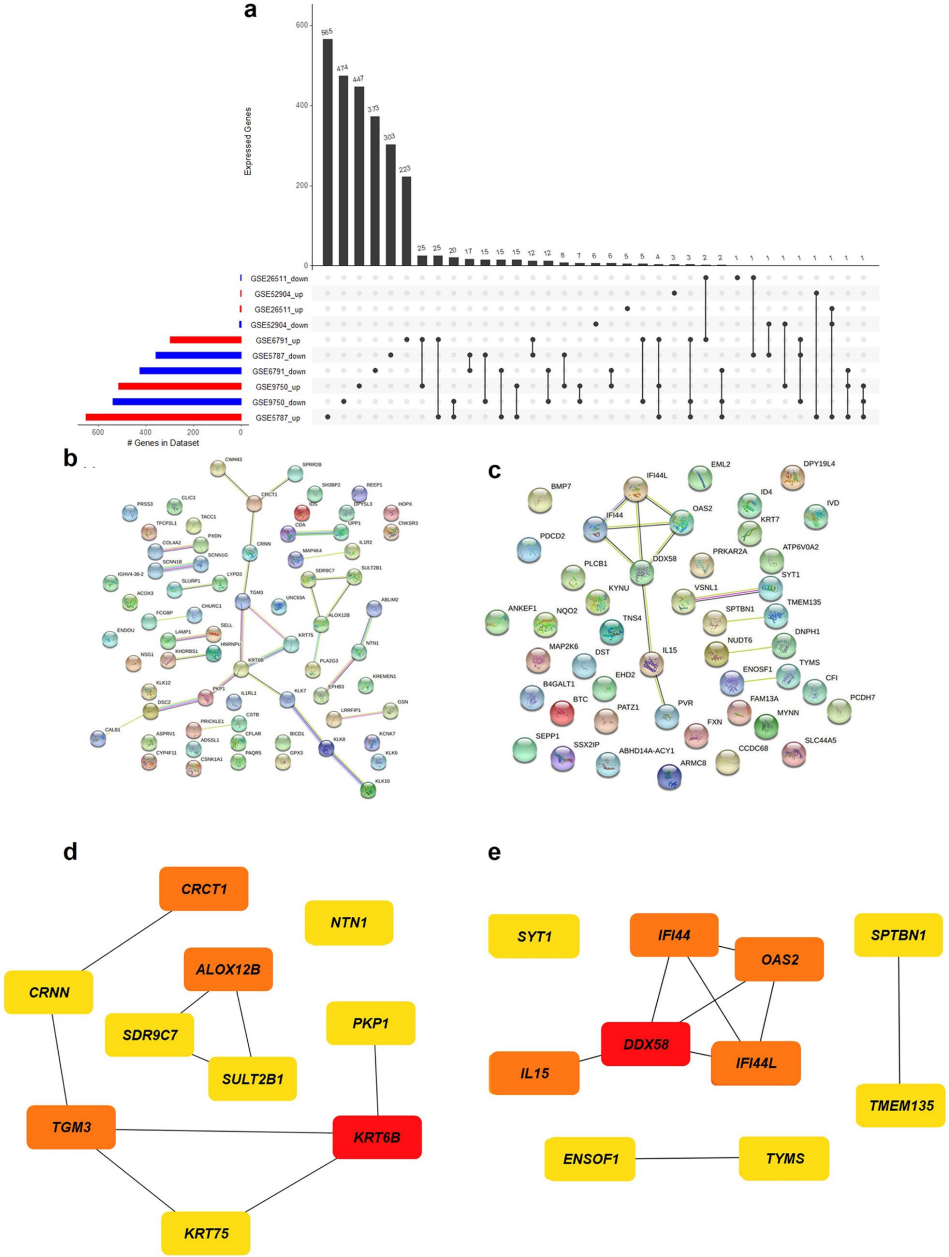
Differential gene expression and pathway analysis **a** An UpSetR plot of DEGs between metastatic and non-metastatic CC tissues retrieved from GEO database (DEGs; with log-fold change > =|1.5| and *p*-value <  = 0.05 cut-off) across five CC studies, **b** Protein–protein interaction network of the upregulated genes, **c** protein–protein interaction network of the downregulated genes, **d** top 10 hub genes upregulated in metastatic CC, **e** top 10 hub genes downregulated in metastatic CC. The hub genes are identified by the highest number of connections in the network. The color scale ranges from yellow (fewer interacting) to red (higher interacting) indicating the relative importance of the hub genes

We performed in silico analysis with the limma R package [216] for identification of differentially expressed genes (DEGs) in five datasets of metastatic cervical cancers deposited in the Gene Expression Omnibus database [215]. Genes with common expression patterns in all the datasets were selected for subsequent analyses. The filtering threshold of *p-value* < 0.05 and *logFoldChange* > 1.5 for upregulation, l*ogFoldChange* < -1.5 for downregulation was set as the standard filter. The genes that fall under the standard filter criteria were selected as DEGs. We have identified 70 upregulated and 46 downregulated genes in at least two datasets. The overlapping DEGs identified in more than two of the selected datasets were extracted and represented in the UpSetR plot Fig. 3a with the UpSetR package [217]. Further, protein–protein interaction (PPI) network was constructed for the overexpressed Fig. 3b and downregulated genes Fig. 3c using Search Tool for the Retrieval of Interacting Genes (STRING) [218]. Additionally, we have used the Maximal Clique Centrality (MCC) algorithm of CytoHubba [219] to identify the hub nodes in the co-expressed network. The densely interacting genes with a score ≥ 0.4 were filtered for the hub genes, and top 10 genes were identified based on the MMC score. Among the hub genes identified with a high MMC score, *KRT6B*, *TGM3*, *ALOX12B*, and *CRCT1* were found to be upregulated Fig. 3d and *DDX58*, *IFI44*, *OAS2*, *IFI44L*, and *IL15* Fig. 3e were downregulated in metastatic CC.

**Fig. 4:**
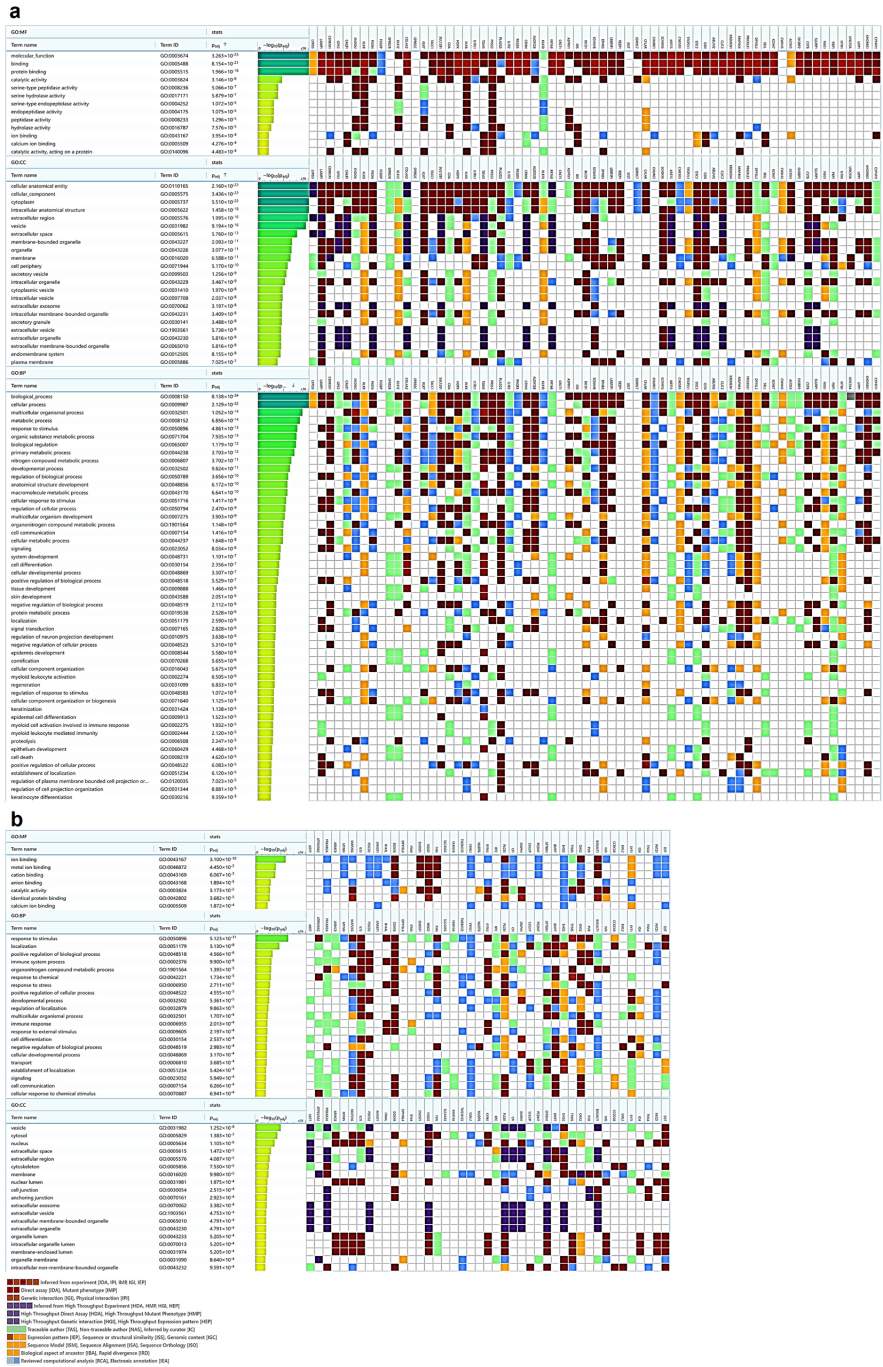
Functional enrichment analysis between metastatic and non-metastatic genes in CC **a** GO terms identification on the upregulated genes in molecular function, biological process and cellular component, **b** GO terms identification on the downregulated genes in molecular function, biological process and cellular component

Further, the gene ontology study identified that the upregulated genes were primarily involved in protein binding and serine-type peptidase activity and the downregulated genes were involved in catalytic activity, localization, and immune response Fig. 4a, b. The functional enrichment analysis of the DEGs was performed for molecular function, biological process, and cellular component using g.Profiler tool [220] with a *p*-value cut-off score of 0.05.

**Fig. 5:**
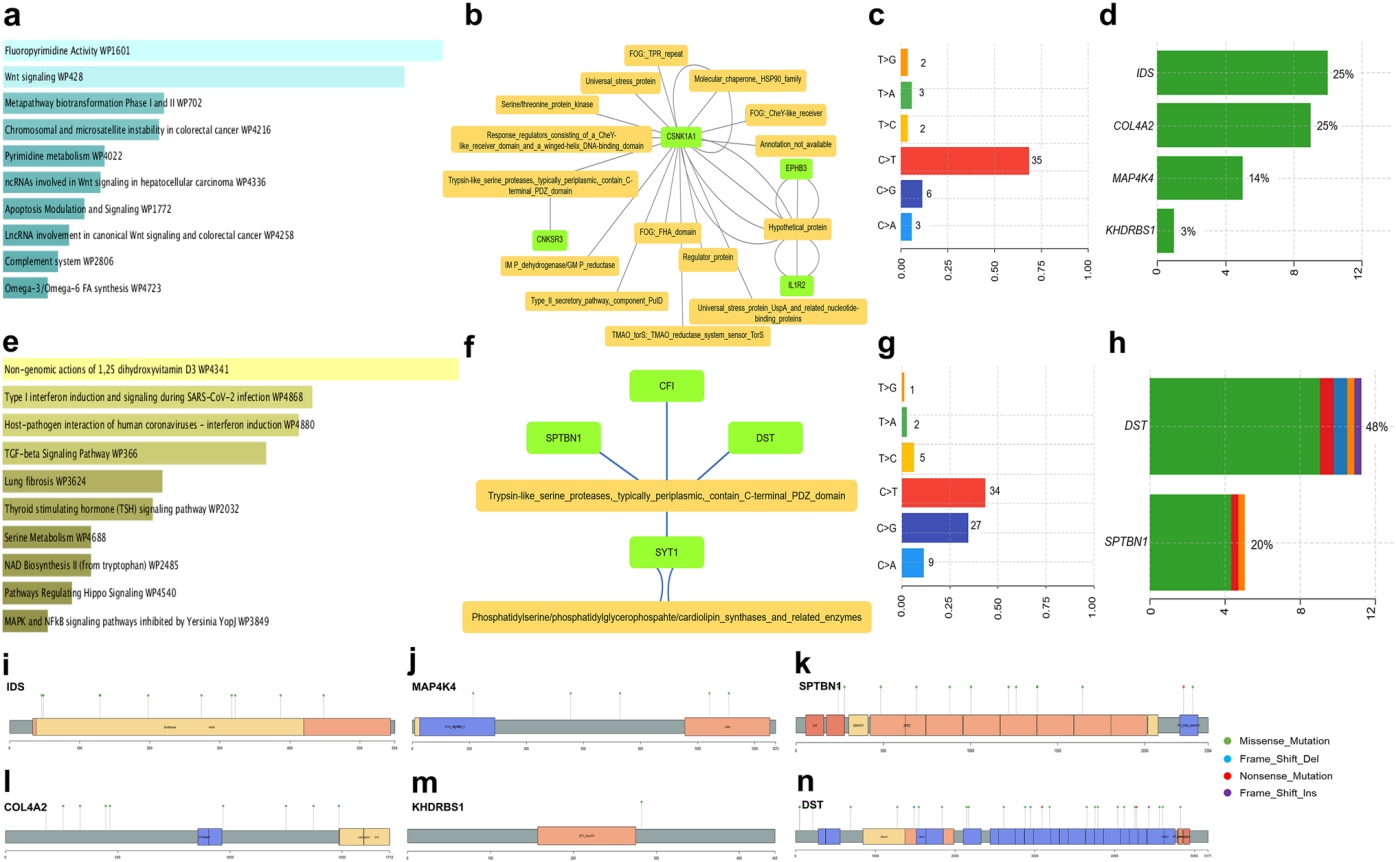
Pathway and network analysis **a** Top ten significant pathways enriched for pathway analysis through WikiPathways in upregulated genes, **b** host–Microbiome protein–domain interaction on upregulated genes, **c** mutation frequency of upregulated genes, **d** mutated genes which are upregulated in metastatic CC, **e** top ten significant pathways enriched for pathway analysis through WikiPathways in downregulated genes, **f** host–microbiome protein–domain interaction on downregulated genes, **g** Mutation frequency of downregulated genes, **h** mutated genes which are downregulated in metastatic CC **i-n** Needle plot for the mutations in IDS, MAP4K4, SPTBN1, COL4A2, KHDRBS1 and DST

When pathway analysis was performed on the DEGs from WikiPathways using EnrichR tool [221] with *p*-value > 0.05 set as the cut-off, suggesting that the upregulated genes were significantly involved in Wnt signaling, chromosomal and microsatellite instability in colorectal cancer, apoptosis modification and signaling Fig. 5a. The downregulated genes showed association with the TGF-β signaling pathway, host–pathogen interaction of human coronavirus-interferon-induced pathways regulating Hippo signaling, MAPK and NF-κB signaling pathways Fig. 5e. Further, using NetworkAnalyst [222], we predicted host–microbiome protein–protein interactions based on the domain–domain binding from the MicrobioLink database [223]. Host–microbiome interaction analysis uncovered four upregulated genes among the DEGs, such as *CNKSR3, CSNK1A1, EPHB3*, and *IL1R2*, interacting with multiple microbial protein domains Fig. 5b and four downregulated genes, *SPTBN1, CFI, DST,* and *SYT1* interacting with two microbial domains Fig. 5f. Mutations in CC dataset from TCGA were obtained for the DEGs and visualized using MAFTools [224]. The mutation frequency for the up- and downregulated genes is shown in Fig. 5c, d and Fig. 5g, h, respectively. The analysis revealed mutations in *IDS, MAP4K4, COL4A2* and *KHDRBS1* among the upregulated and, in *SPTBN1* and *DST* among the downregulated genes Fig. 5i–n.

**Fig. 6:**
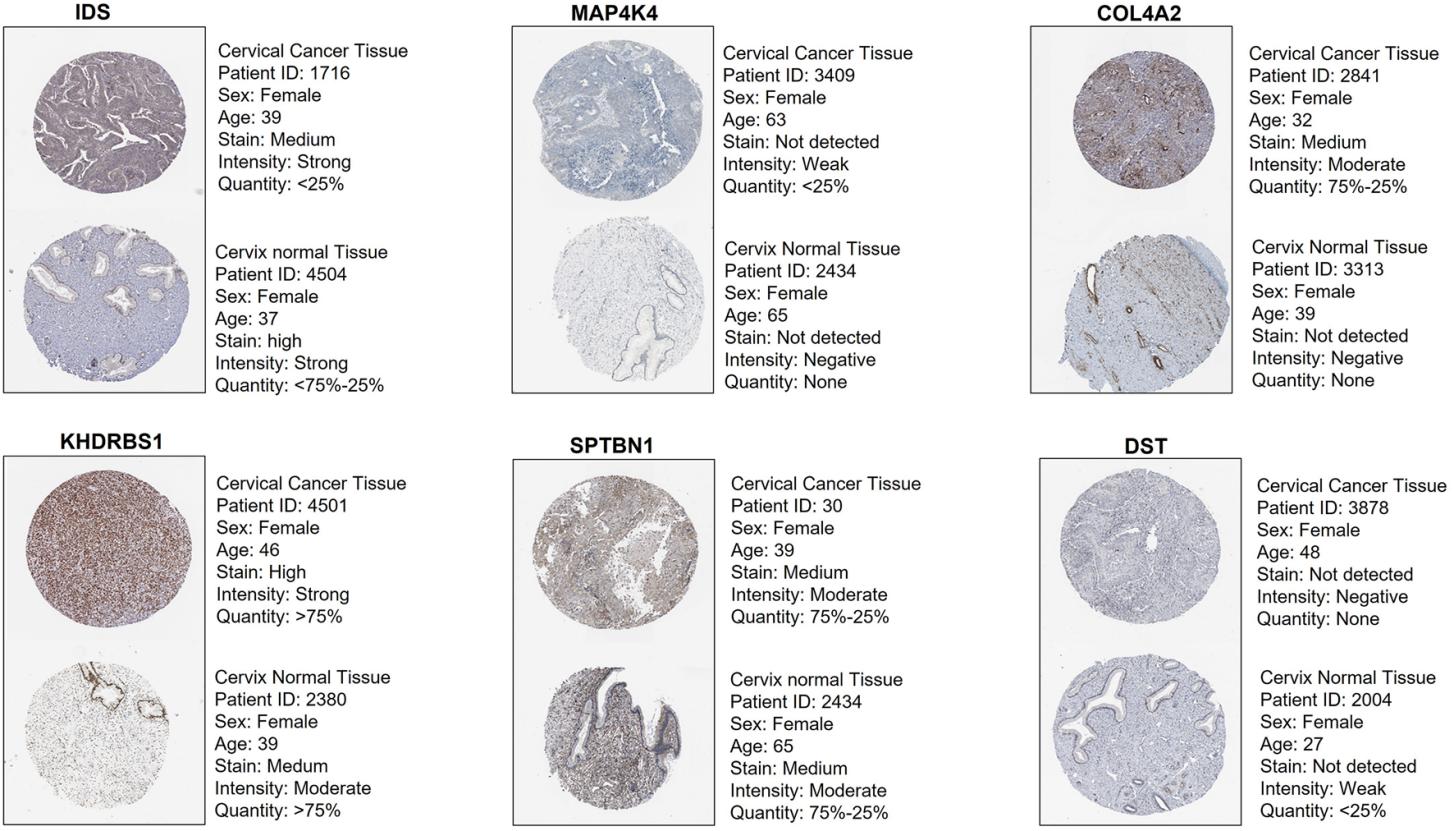
Immunohistochemistry image representation from Human Protein Atlas for IDS, MAP4K4, COL4A2, KHDRBS1, showing significant upregulation in CC and downregulation in normal cervix tissue. SPTBN1 and DST showed downregulation or not detected in CC with concurrent upregulation in normal tissue

Protein expression analysis was performed for DEGs using Human Protein Atlas [225], revealing significant difference in the expression of IDS, MAP4K4, COL4A2, KHDRBS1 in CC and normal cervical epithelial tissues Fig. 6. *IDS* encoding iduronate 2-sulfatase (ID2S) was overexpressed in CC epithelial tissue compared to the normal tissue with an unknown mutation status. ID2S is primarily reported in mucopolysaccharidosis, and its association with the regulation of breast cancer metastasis has been reported [226, 227]. MAP4K4 is a threonine/serine kinase [228] that plays a significant role in a variety of physiological processes, including embryonic development, immunological response, inflammation, insulin sensitivity, and metabolic diseases [229–232]. Overexpression of *MAP4K4* is associated with the activation of NF-κB and JNK signaling pathway and EMT induction in hepatocellular carcinoma (HCC) [233]. Studies have also shown that SOX6 is a downstream target of MAPK4 that triggers autophagy in CC and it functions through the inhibition of the PI3K-AKT-mTOR pathway and activation of the MAPK/ERK pathway [234]. *MAP4K4* is reported as one of the potential metastatic genes in colorectal cancer metastasis [235] and CC [236]. Downregulation of *MAP4K4* in cancer cell lines inhibits cell proliferation and cell growth [237, 238], apoptosis induction [239, 240], and migration and invasion [241].

*COL4A2* encodes type IV collagen and impacts cancer progression and pathogenesis. In HCC, *COL4A1* overexpression promotes metastasis [242]. *COL4A1* overexpression is positively correlated with dendritic cells and macrophage expression in CC [242]. Suppression of *COL4A2* inhibits migration and proliferation in triple-negative breast cancer [243]. Elevated expression of *KHDRBS1/SAM68* detected in CC tissues with pelvic node metastasis and was not found in normal tissues [244]. The RNA-binding protein (RBP), SRC associated in mitosis of 68 kDa (SAM68), is a member of signal transduction and activation of RNA (STAR) family. It contains a functional RNA-binding KH domain and binds to single-stranded nucleic acids. During mitosis, SAM68 is tyrosine-phosphorylated and associated with Src. The tyrosine phosphorylation mediates the interactions with SH2- and SH3-containing proteins. It participates in cell growth and differentiation [245]. SAM68 is reported to play a dual role in cancer, i.e., tumor suppressor as well as an oncogene. Under the influence of extracellular stimuli, the level of SAM68 expression and phosphorylation influences tumor progression. For example, SAM68 phosphorylation inhibited cell proliferation induced by breast tumor kinase (BRK) [246]. SAM68 expression is elevated in CC, and its cytoplasmic location is linked to LNM. EMT induction is mediated via AKT/ GSK-3/SNAIL when SAM68 is downregulated, indicating its oncogenic function in cervical cancer [244]. Further, SAM68 expression levels were elevated in non-melanoma skin cancer tissue samples, and the increased expression was associated with enhanced growth and proliferation of A431 cells, while knocking down of *SAM68* in malignant keratinocytes increases the sensitivity to DNA damage and decreases tumor burden [247].

SPTBN1 is a member of the β spectrin family and is important for the stability of lateral membrane. It also functions as a TGF-β adaptor protein and modulates Smad complex [248]. SPTBN1 has been associated with several malignancies, and its expression varies according to the stage and type of cancer. *SPTBN1* expression decreased significantly with the progression of epithelial ovarian cancer. The decreased expression resulted in cell proliferation, migration, and increased vimentin expression [249]. It inhibited the progression of ovarian cancer via the JAK/STAT3 pathway. Its expression was much lower in more invasive ovarian cancer cell lines, suggesting that it may play a role in tumor aggressiveness [249]. Further, the BAP31/SPTBN1 complex was found to regulate CC progression via the TGF-β/ SMAD signaling pathway under the regulation of miR-362 [250]. In primary breast cancer tissues, the SPTBN1 expression was shown to be considerably downregulated as compared to normal, and it was identified to be a potent inhibitor of EMT and breast cancer progression [251]. Similarly, *SPTBN1* is downregulated in HCC and loss of its expression stimulates Wnt signaling, thereby promoting the acquisition of stem cell-like characteristics, and eventually, contributing to the growth of malignant tumors [252].

Further, analysis from two datasets (GSE6791 and GSE9750), various isoforms of kallikreins (KLKs) -*KLK8, KLK7, KLK10*) were found upregulated in metastasized CC. Kallikrein (KLK) proteins are a subgroup of serine proteases. KLKs play an important role in carcinogenesis by altering cell proliferation, EMT, compromising oxygen balance, and degrading ECM. They also allow tumor cell detachment by infiltrating through ECM barriers and promoting metastatic dissemination [253]. Kallikrein-related peptidase 8 (KLK8) is one such protein that has been identified to be highly expressed in cervical, ovarian and endometrial cancers, indicating that it could be a viable target for therapy [254, 255]. KLK8 induced colorectal cancer development by triggering EMT [256]. The wound healing and Transwell migration assays revealed that KLK8 promoted cell proliferation, invasion and metastasis [256]. The upregulation was also associated with metastasis of head and neck SCC to cervical lymph nodes [257]. Further, it was identified to be significantly downregulated in breast cancer tissues [258]. Though the mechanisms of metastasis progression are not fully understood, it can degrade collagen type IV and fibronectin, both of which are required for cancer invasion and metastasis [259].

In CC tissues and serum, kallikrein 7 (*KLK7*) levels were reported to be higher, both of which are associated with tumor growth, invasion and metastasis [260, 261]. *KLK7* was found to be regulated by histone modifications and promoter methylation. Trichostatin A (TSA) enhanced *KLK7* expression in cervical and pancreatic cancer cell lines, and the transcription factor specificity protein 1 (SP1) supported the effect of TSA on *KLK7* by transcriptional activation [262]. KLK7 facilitates various processes involved in cancer, such as cell growth, proliferation, migration, angiogenesis via hydrolyzing cytokines, ECM, and membrane proteins [263]. Overexpression of *KLK7* in pancreatic cancer was associated with urokinase-type plasminogen activator receptor (uPAR), which inhibited cellular adherence to vitronectin [264]. Further, in melanoma, *KLK7* overexpression inhibited cell adhesion by lowering integrin expression and inducing spheroid formation by increasing MCAM/CD146 expression [190]. The expression in oral SCC was similarly shown to increase as the malignancy progressed from stage I to stage IV [265]. The brain cancer cells also showed high *KLK7* expression, which significantly induced the invasion and metastasis [266].

KLK10 is another member of serine protease family closely related to glandular kallikreins [267]. The gene was found to be downregulated in breast and prostate cancer cell lines, and tumor growth was inhibited in vivo upon its overexpression, indicating its tumor-suppressor function [268]. Its expression is regulated via CpG hypermethylation in ovarian, breast, prostate cancer as well as in acute lymphoblastic leukemia [269, 270]. Further, overexpression of *KLK10* reduced the proliferation and invasive potential of tongue cancer cells [271]. Metastasis-associated protein 2 (MTA2) has been linked to metastasis and tumor growth. Downregulation of *MTA2* prevented migration and invasion while increasing *KLK10* expression via SP1 transcription factor in CC cell lines [272]. *KLK10*, on the other hand, was found to be upregulated in colorectal cancer, the loss of expression of which induced apoptosis, caspase 3 activity, and inhibited proliferation via the PI3K/AKT/mTOR signaling pathway [123].

Protocadherins (PCDH) belong to the non-classical subfamily of cadherins. These are highly expressed in brain and mediate cell to cell interactions [273]. Several studies have reported that PCDH is abnormally expressed in numerous malignancies and show either a carcinogenic or anti-tumor effect [274], suggesting distinct family members with effects that are tumor dependent. *PCDH7* was found to be overexpressed in NSCLC and reported to show oncogenic activity by hyperactivating MAPK pathway [275]. *PCDH7* expression was also shown to be upregulated in prostate cancer cells [276]. Colony formation, migration, and invasion were all impeded by the loss of its expression. It may play a role in the activation of the AKT and ERK pathways during the advancement of prostate cancer [276]. The expression was reduced in gastric cancer, particularly in cases of LNM. Cell migration, invasion, and E-cadherin expression were all considerably reduced because of *PCDH7* downregulation [277]. Furthermore, in CC, decreased expression of *PCDH7* was linked to cancer cell metastasis, migration and invasion [278]. The role of MAP4K4, SPTBN1, PCDH7, KLK7, KLK8, KLK10 and SAM68, which were identified to be differentially expressed among metastatic compared to non-metastatic CC in our bioinformatic analysis and their effect on the metastatic progression of various other type of cancer is summarized in Table 2.

**Table 2:** Evidence for the role of shortlisted differentially regulated genes on the metastatic progression of various cancer types

Protein (Expression status)	Cancer type	Observation	Reference
MAP4K4 (Upregulated in cancer)	Hepatocellular carcinoma	EMT, migration to lungs, proliferation, invasion, activate JNK, NF-κβ pathway	[233]
	Cervical cancer	Induces autophagy by inhibiting PI3K-AKT-mTOR pathway and activate MAPK/ERK pathway	[234]
	B cell lymphoma	Prevent apoptosis, induces EMT	[279]
	Pancreatic cancer (PDAC)	Cell proliferation	[280]
SPTBN1 (Upregulated in cancer)	Breast cancer	Prevent EMT and breast cancer proliferation	[251]
	Hepatocellular carcinoma	Activates Wnt signaling and promotes stem cell like features	[252]
	Ovarian cancer	SPTBN1 activates the JAK/STAT3 and mesenchymal transformation	[249]
	Cervical cancer	SPTBN1 activates TGF-β/ Smad pathway to exert anti-tumor effect	[250]
PCDH7 (Downregulated in cancer)	Cervical cancer	Down regulated in cervical cancer contributes to migration and invasion	[278]
	Gastric cancer	Cell migration and invasion is inhibited through E-cadherin	[277]
KLK8 (Upregulated in cancer)	Cervical cancer	Immunohistochemistry staining conformed TADG-14 overexpression in cervical cancer specimens	[254]
	Colorectal cancer	Promote EMT and CRC progression effect is mediated PAR1-dependent pathway	[256]
	Cervical cancer	KLK8 expression is correlated with the precancerous and cancerous stages of HPV infection	[281]
KLK7 (Upregulated in cancer)	Cervical cancer	Correlated lymph node metastasis, and stromal invasion	[260]
	Melanoma	Switch from proliferative to invasive phenotype, downregulation of cell adhesion molecules, homotypic cell adhesion and interaction with the extracellular matrix	[282]
	Prostate cancer	Promotes invasion and metastasis through inducing EMT	[283]
KLK10 (Downregulated in cancer)	Cervical cancer	miR-199 b-5p on promoting cervical cancer progression by inhibiting KLK10 expression	[284]
	Prostate cancer	Downregulation of Bcl-2 promotes apoptosis	[285]
SAM68 (Upregulated in cancer)	Cervical cancer	EMT, pelvic lymph node metastasis in cervical cancer patients	[244]
	Breast cancer	Lymph node metastasis and cancer progression	[286]

## Conclusion

The pathogenesis of CC is intricate and interconnected to the key determinants of molecular interactions and biological effects. HPV infection along with other risk factors is known to initiate the CC by recreating aberrant local microenvironment. Further, genetic and epigenetic modifications along with altered gene expression may favor the progression and metastasis of the cancer cells. The role of microbiota is significant in the modulation of microenvironment to facilitate the infection by HPV and also to promote metastasis of the tumor. Hence, understanding and elucidating the functional properties of the diverse species in the cervix will aid in deciphering the role of cervical microbiota (viral, fungal and bacterial) in the progression of CIN to CC and metastasis. Sentinel lymph nodes are the first nodes to receive lymphatic drainage from the primary lesion and promote LNM due to immune suppression by the tumor microenvironment. Further, lymphatic system can facilitate metastasis to distant organs, such as bone, lung, liver, and brain. Hence, identification of LNM and modulation of its microenvironment is required to prevent metastasis to distant organs and to provide effective treatment. Number of molecular pathways and their downstream functions are altered because of viral proteins, HPV integration, somatic mutation, copy number variation, epigenetic alteration, differential expression of the genes, miRNAs, lncRNAs and circRNAs, and interestingly, each of these factors is interconnected such that they favor incremental evolution, development and progression of CC. Implication of multi-omics approach to determine the genomic landscape can aid the stratification of CC patients and formulating effective treatment strategies.

## Data Availability

Enquiries about data availability should be directed to the authors.

## References

[1] Zhang T, Zou P, Wang T (2016). Down-regulation of miR-320 associated with cancer progression and cell apoptosis via targeting Mcl-1 in cervical cancer. Tumour Biol.

[2] Sung H, Ferlay J, Siegel RL (2021). Global cancer statistics 2020: GLOBOCAN estimates of Incidence and mortality worldwide for 36 cancers in 185 countries. CA Cancer J Clin.

[3] Guo H, Wang S, Ju M (2021). Identification of stemness-related genes for cervical squamous cell carcinoma and endocervical adenocarcinoma by integrated bioinformatics analysis. Front Cell Dev Biol.

[4] Siegel R, Ward E, Brawley O, Jemal A (2011). Cancer statistics, 2011: the impact of eliminating socioeconomic and racial disparities on premature cancer deaths. CA Cancer J Clin.

[5] Aziz SW, Aziz MH, Ahmad A (2017). Cervical Cancer Metastasis. Introduction to Cancer Metastasis.

[6] Tulay P, Serakinci N (2016). The role of human papillomaviruses in cancer progression. J Cancer Metastasis Treat.

[7] Wu Z, Yu L, Lei X (2018). The association between human papillomavirus 16, 18 DNA load and E6 protein expression in cervical intraepithelial neoplasia and cancer. J Clin Virol.

[8] Da S, Pasumarthi D, Pasha A (2021). Identification of differentially expressed genes in cervical cancer patients by comparative transcriptome analysis. Biomed Res Int.

[9] Liu JJ, Ho JY, Lee JE (2020). Genomic, transcriptomic, and viral integration profiles associated with recurrent/metastatic progression in high-risk human papillomavirus cervical carcinomas. Cancer Med.

[10] Xu Y, Luo H, Hu Q, Zhu H (2021). Identification of potential driver genes based on multi-genomic data in cervical cancer. Front Genet.

[11] Tornesello ML, Faraonio R, Buonaguro L (2020). The Role of microRNAs, Long Non-coding RNAs, and Circular RNAs in Cervical Cancer. Front Oncol.

[12] Wilting SM, Snijders PJ, Verlaat W (2013). Altered microRNA expression associated with chromosomal changes contributes to cervical carcinogenesis. Oncogene.

[13] Junttila MR, de Sauvage FJ (2013). Influence of tumour micro-environment heterogeneity on therapeutic response. Nature.

[14] Perea Paizal J, Au SH, Bakal C (2021). Squeezing through the microcirculation: survival adaptations of circulating tumour cells to seed metastasis. Br J Cancer.

[15] Zeeshan R, Mutahir Z (2017). Cancer metastasis - tricks of the trade. Bosn J Basic Med Sci.

[16] Welch DR, Hurst DR, Coleman WB, Tsongalis GJ (2017). Beyond the primary tumor: progression, invasion, and metastasis. The molecular basis of human cancer.

[17] Talmadge JE, Fidler IJ (2010). AACR centennial series: the biology of cancer metastasis: historical perspective. Cancer Res.

[18] Castanheira CP, Sallas ML, Nunes RAL, Lorenzi NPC, Termini L (2021). Microbiome and cervical cancer. Pathobiology.

[19] Fidler IJ (1985). Macrophages and metastasis-a biological approach to cancer therapy. Cancer Res.

[20] Estrella V, Chen T, Lloyd M (2013). Acidity generated by the tumor microenvironment drives local invasion. Cancer Res.

[21] Hagemann T, Bozanovic T, Hooper S (2007). Molecular profiling of cervical cancer progression. Br J Cancer.

[22] MacDonald BT, Tamai K, He X (2009). Wnt/beta-catenin signaling: components, mechanisms, and diseases. Dev Cell.

[23] Massague J (2008). TGFbeta in Cancer. Cell.

[24] Chikazawa N, Tanaka H, Tasaka T (2010). Inhibition of Wnt signaling pathway decreases chemotherapy-resistant side-population colon cancer cells. Anticancer Res.

[25] Shinohara A, Yokoyama Y, Wan X (2001). Cytoplasmic/nuclear expression without mutation of exon 3 of the beta-catenin gene is frequent in the development of the neoplasm of the uterine cervix. Gynecol Oncol.

[26] Chen J, Qiu J, Li F (2020). HN1 promotes tumor associated lymphangiogenesis and lymph node metastasis via NF-kappaB signaling activation in cervical carcinoma. Biochem Biophys Res Commun.

[27] Klein C, Kahesa C, Mwaiselage J, West JT, Wood C, Angeletti PC (2020). How the cervical microbiota contributes to cervical cancer risk in Sub-Saharan Africa. Front Cell Infect Microbiol.

[28] Sakamoto J, Kamiura S, Okayama K (2018). Single type infection of human papillomavirus as a cause for high-grade cervical intraepithelial neoplasia and invasive cancer in Japan. Papillomavirus Res.

[29] Deligeoroglou E, Giannouli A, Athanasopoulos N (2013). HPV infection: immunological aspects and their utility in future therapy. Infect Dis Obstet Gynecol.

[30] Steinbach A, Riemer AB (2018). Immune evasion mechanisms of human papillomavirus: An update. Int J Cancer.

[31] Tornesello ML, Annunziata C, Tornesello AL (2018). Human oncoviruses and p53 tumor suppressor pathway deregulation at the origin of human cancers. Cancers (Basel).

[32] Yeo-Teh NSL, Ito Y, Jha S (2018). High-risk human papillomaviral oncogenes E6 and E7 target key cellular pathways to achieve oncogenesis. Int J Mol Sci.

[33] Tilborghs S, Corthouts J, Verhoeven Y (2017). The role of nuclear factor-kappa B signaling in human cervical cancer. Crit Rev Oncol Hematol.

[34] Panczyszyn A, Boniewska-Bernacka E, Glab G (2020). Telomere length in leukocytes and cervical smears of women with high-risk human papillomavirus (HR HPV) infection. Taiwan J Obstet Gynecol.

[35] Veldman T, Horikawa I, Barrett JC, Schlegel R (2001). Transcriptional activation of the telomerase hTERT gene by human papillomavirus type 16 E6 oncoprotein. J Virol.

[36] Liu G, Sharma M, Tan N (2018). HIV-positive women have higher risk of human papilloma virus infection, precancerous lesions, and cervical cancer. AIDS.

[37] Klein C, Gonzalez D, Samwel K (2019). Relationship between the cervical microbiome, HIV status, and precancerous lesions. MBio.

[38] Chen T, Yang S, Xu J (2020). Transcriptome sequencing profiles of cervical cancer tissues and SiHa cells. Funct Integr Genomics.

[39] Gillet E, Meys JF, Verstraelen H (2012). Association between bacterial vaginosis and cervical intraepithelial neoplasia: systematic review and meta-analysis. PLoS ONE.

[40] Uhlorn BL, Jackson R, Li S (2020). Vesicular trafficking permits evasion of cGAS/STING surveillance during initial human papillomavirus infection. PLoS Pathog.

[41] Prati B, Marangoni B, Boccardo E (2018). Human papillomavirus and genome instability: from productive infection to cancer. Clinics (Sao Paulo).

[42] Kemp TJ, Hildesheim A, Garcia-Pineres A (2010). Elevated systemic levels of inflammatory cytokines in older women with persistent cervical human papillomavirus infection. Cancer Epidemiol Biomarkers Prev.

[43] Liebenberg LJP, McKinnon LR, Yende-Zuma N (2019). HPV infection and the genital cytokine milieu in women at high risk of HIV acquisition. Nat Commun.

[44] Fernandes JV (2015). Link between chronic inflammation and human papillomavirus-induced carcinogenesis (Review). Oncol Lett.

[45] Liang WS, Aldrich J, Nasser S (2014). Simultaneous characterization of somatic events and HPV-18 integration in a metastatic cervical carcinoma patient using DNA and RNA sequencing. Int J Gynecol Cancer.

[46] Wira CR, Grant-Tschudy KS, Crane-Godreau MA (2005). Epithelial cells in the female reproductive tract: a central role as sentinels of immune protection. Am J Reprod Immunol.

[47] Balasubramaniam SD, Balakrishnan V, Oon CE, Kaur G (2019). Key molecular events in cervical cancer development. Medicina (Kaunas).

[48] Chalifour AA, Poveda KT, Roman BM (2016). Cervical microbiome and cytokine profile at various stages of cervical cancer: a pilot study. PLoS ONE.

[49] Rubinstein MR, Wang X, Liu W, Hao Y, Cai G, Han YW (2013). Fusobacterium nucleatum promotes colorectal carcinogenesis by modulating E-cadherin/β-catenin signaling via its FadA adhesin. Cell Host Microbe.

[50] Fidler IJ, Radinsky R (1990). Genetic control of cancer metastasis. J Natl Cancer Inst.

[51] Nguyen DX, Massague J (2007). Genetic determinants of cancer metastasis. Nat Rev Genet.

[52] Stupack DG, Teitz T, Potter MD (2006). Potentiation of neuroblastoma metastasis by loss of caspase-8. Nature.

[53] Gupta GP, Nguyen DX, Chiang AC (2007). Mediators of vascular remodelling co-opted for sequential steps in lung metastasis. Nature.

[54] Xu T, Zeng Y, Shi L (2020). Targeting NEK2 impairs oncogenesis and radioresistance via inhibiting the Wnt1/beta-catenin signaling pathway in cervical cancer. J Exp Clin Cancer Res.

[55] Li H, Zhang W, Yan M (2019). Nucleolar and spindle associated protein 1 promotes metastasis of cervical carcinoma cells by activating Wnt/beta-catenin signaling. J Exp Clin Cancer Res.

[56] Gao X, Chen G, Cai H (2020). Aberrantly enhanced melanoma-associated antigen (MAGE)-A3 expression facilitates cervical cancer cell proliferation and metastasis via actuating Wnt signaling pathway. Biomed Pharmacother.

[57] Xu J, Lu W (2020). FAM83A exerts tumorsuppressive roles in cervical cancer by regulating integrins. Int J Oncol.

[58] Chen C, Li HF, Hu YJ, Jiang MJ (2019). Family with sequence similarity 83 member H promotes the viability and metastasis of cervical cancer cells and indicates a poor prognosis. Yonsei Med J.

[59] Zhang X, Liu S, Zhu Y (2020). A-kinase-interacting protein 1 promotes EMT and metastasis via PI3K/Akt/IKKbeta pathway in cervical cancer. Cell Biochem Funct.

[60] Zhu B, Liu Q, Han Q (2018). Downregulation of Kruppellike factor 1 inhibits the metastasis and invasion of cervical cancer cells. Mol Med Rep.

[61] Li J, Qi C, Liu X, Li C, Chen J, Shi M (2018). Fibulin-3 knockdown inhibits cervical cancer cell growth and metastasis in vitro and in vivo. Sci Rep.

[62] Che Y, Li Y, Zheng F (2019). TRIP4 promotes tumor growth and metastasis and regulates radiosensitivity of cervical cancer by activating MAPK, PI3K/AKT, and hTERT signaling. Cancer Lett.

[63] Wu G, Long Y, Lu Y (2020). Kindlin2 suppresses cervical cancer cell migration through AKT/mTORmediated autophagy induction. Oncol Rep.

[64] Cao M, Gao D, Zhang N (2019). Shp2 expression is upregulated in cervical cancer, and Shp2 contributes to cell growth and migration and reduces sensitivity to cisplatin in cervical cancer cells. Pathol Res Pract.

[65] Jiang C, Xu R, Li XX (2017). p53R2 overexpression in cervical cancer promotes AKT signaling and EMT, and is correlated with tumor progression, metastasis and poor prognosis. Cell Cycle.

[66] Zheng J, Dai X, Chen H (2018). Down-regulation of LHPP in cervical cancer influences cell proliferation, metastasis and apoptosis by modulating AKT. Biochem Biophys Res Commun.

[67] Zhang C, Liao Y, Liu P (2020). FABP5 promotes lymph node metastasis in cervical cancer by reprogramming fatty acid metabolism. Theranostics.

[68] Cai H, Yan L, Liu N (2020). IFI16 promotes cervical cancer progression by upregulating PD-L1 in immunomicroenvironment through STING-TBK1-NF-kB pathway. Biomed Pharmacother.

[69] Yang Y, Han A, Wang X (2021). Lipid metabolism regulator human hydroxysteroid dehydrogenase-like 2 (HSDL2) modulates cervical cancer cell proliferation and metastasis. J Cell Mol Med.

[70] Wu H, Song S, Yan A (2019). RACK1 promotes the invasive activities and lymph node metastasis of cervical cancer via galectin-1. Cancer Lett.

[71] Chetry M, Song Y, Pan C (2020). Effects of galectin-1 on biological behavior in cervical cancer. J Cancer.

[72] Deng M, Cai X, Long L (2019). CD36 promotes the epithelial-mesenchymal transition and metastasis in cervical cancer by interacting with TGF-beta. J Transl Med.

[73] Zou R, Chen X, Jin X (2018). Up-regulated BCAR4 contributes to proliferation and migration of cervical cancer cells. Surg Oncol.

[74] Jiang Y, Ren W, Wang W (2017). Inhibitor of beta-catenin and TCF (ICAT) promotes cervical cancer growth and metastasis by disrupting E-cadherin/beta-catenin complex. Oncol Rep.

[75] Li J, Liu C, Li D (2019). OLFM4 inhibits epithelial-mesenchymal transition and metastatic potential of cervical cancer cells. Oncol Res.

[76] Xu L, Li C, Hua F (2021). The CXCL12/CXCR7 signalling axis promotes proliferation and metastasis in cervical cancer. Med Oncol.

[77] Ma H, Han F, Yan X (2021). PBK promotes aggressive phenotypes of cervical cancer through ERK/c-Myc signaling pathway. J Cell Physiol.

[78] Li Y, Li S, Huang L (2018). Knockdown of Rap2B, a Ras Superfamily Protein, Inhibits Proliferation, Migration, and Invasion in Cervical Cancer Cells via Regulating the ERK1/2 Signaling Pathway. Oncol Res.

[79] Zhan F, Zhong Y, Qin Y (2020). SND1 facilitates the invasion and migration of cervical cancer cells by Smurf1-mediated degradation of FOXA2. Exp Cell Res.

[80] Yang L, Yu Y, Xiong Z (2020). Downregulation of SEMA4C Inhibit epithelial-mesenchymal transition (EMT) and the invasion and metastasis of cervical cancer cells via inhibiting transforming growth factor-beta 1 (TGF-beta1)-Induced hela cells p38 mitogen-activated protein kinase (MAPK) activation. Med Sci Monit.

[81] Xu LX, Hao LJ, Ma JQ (2020). SIRT3 promotes the invasion and metastasis of cervical cancer cells by regulating fatty acid synthase. Mol Cell Biochem.

[82] Zhu L, Yu CL, Zheng Y (2019). NSD2 inhibition suppresses metastasis in cervical cancer by promoting TGF-beta/TGF-betaRI/SMADs signaling. Biochem Biophys Res Commun.

[83] Zeng YT, Liu XF, Yang WT (2019). REX1 promotes EMT-induced cell metastasis by activating the JAK2/STAT3-signaling pathway by targeting SOCS1 in cervical cancer. Oncogene.

[84] Mao Z, Sang MM, Chen C, Zhu WT (2019). CSN6 promotes the migration and invasion of cervical cancer cells by inhibiting autophagic degradation of cathepsin L. Int J Biol Sci.

[85] Tian T, Li X, Hua Z (2017). S100A7 promotes the migration, invasion and metastasis of human cervical cancer cells through epithelial-mesenchymal transition. Oncotarget.

[86] Hou C, Zhuang Z, Deng X (2018). Knockdown of trio by CRISPR/Cas9 suppresses migration and invasion of cervical cancer cells. Oncol Rep.

[87] Wang J, Feng Y, Chen X (2018). SH3BP1-induced Rac-Wave2 pathway activation regulates cervical cancer cell migration, invasion, and chemoresistance to cisplatin. J Cell Biochem.

[88] Pang T, Li M, Zhang Y (2017). Y box-binding protein 1 promotes epithelial-mesenchymal transition, invasion, and metastasis of cervical cancer via enhancing the expressions of snail. Int J Gynecol Cancer.

[89] Xi M, Tang W (2020). Knockdown of Ezrin inhibited migration and invasion of cervical cancer cells in vitro. Int J Immunopathol Pharmacol.

[90] Chen G, Yu X, Zhang M (2019). Inhibition of euchromatic histone lysine methyltransferase 2 (EHMT2) suppresses the proliferation and invasion of cervical cancer cells. Cytogenet Genome Res.

[91] Su HC, Wu SC, Yen LC (2020). Gene expression profiling identifies the role of Zac1 in cervical cancer metastasis. Sci Rep.

[92] Kurmyshkina OV, Belova LL, Kovchur PI (2015). Remodeling of angiogenesis and lymphangiogenesis in cervical cancer development. Biomed Khim.

[93] Alitalo A, Detmar M (2012). Interaction of tumor cells and lymphatic vessels in cancer progression. Oncogene.

[94] Paduch R (2016). The role of lymphangiogenesis and angiogenesis in tumor metastasis. Cell Oncol (Dordr).

[95] Shibuya M (2011). Vascular endothelial growth factor (VEGF) and its receptor (VEGFR) signaling in angiogenesis: a crucial target for anti- and pro-angiogenic therapies. Genes Cancer.

[96] Vogelstein B, Papadopoulos N, Velculescu VE (2013). Cancer genome landscapes. Science.

[97] Burrell RA, McGranahan N, Bartek J (2013). The causes and consequences of genetic heterogeneity in cancer evolution. Nature.

[98] Bhatla N, Berek JS, Cuello Fredes M (2019). Revised FIGO staging for carcinoma of the cervix uteri. Int J Gynaecol Obstet.

[99] Olthof EP, van der Aa MA, Adam JA (2021). The role of lymph nodes in cervical cancer: incidence and identification of lymph node metastases-a literature review. Int J Clin Oncol.

[100] Levenback C, Coleman RL, Burke TW (2002). Lymphatic mapping and sentinel node identification in patients with cervix cancer undergoing radical hysterectomy and pelvic lymphadenectomy. J Clin Oncol.

[101] Bader AA, Winter R, Haas J (2007). Where to look for the sentinel lymph node in cervical cancer. Am J Obstet Gynecol.

[102] Van Trappen PO, Steele D, Lowe DG (2003). Expression of vascular endothelial growth factor (VEGF)-C and VEGF-D, and their receptor VEGFR-3, during different stages of cervical carcinogenesis. J Pathol.

[103] Jach R, Dulinska-Litewka J, Laidler P (2010). Expression of VEGF, VEGF-C and VEGFR-2 in in situ and invasive SCC of cervix. Front Biosci (Elite Ed).

[104] Cimpean AM, Mazuru V, Cernii A (2011). Detection of early lymphangiogenesis by lymphatic microvascular density and endothelial proliferation status in preneoplastic and neoplastic lesions of the uterine cervix. Pathol Int.

[105] Shen GH, Ghazizadeh M, Kawanami O (2000). Prognostic significance of vascular endothelial growth factor expression in human ovarian carcinoma. Br J Cancer.

[106] Jalkanen S, Salmi M (2020). Lymphatic endothelial cells of the lymph node. Nat Rev Immunol.

[107] Breslin JW, Kurtz KM (2009). Lymphatic endothelial cells adapt their barrier function in response to changes in shear stress. Lymphat Res Biol.

[108] Zhang L, Chen Q, Hu J (2016). Expression of HIF-2alpha and VEGF in cervical squamous cell carcinoma and its clinical significance. Biomed Res Int.

[109] Zhang L, Chen Q, Hu J (2017). Corrigendum to "expression of HIF-2alpha and VEGF in cervical squamous cell carcinoma and its clinical significance". Biomed Res Int.

[110] Li K, Sun H, Lu Z (2018). Value of [^18^F]FDG PET radiomic features and VEGF expression in predicting pelvic lymphatic metastasis and their potential relationship in early-stage cervical squamous cell carcinoma. Eur J Radiol.

[111] Hsieh SH, Ying NW, Wu MH (2008). Galectin-1, a novel ligand of neuropilin-1, activates VEGFR-2 signaling and modulates the migration of vascular endothelial cells. Oncogene.

[112] Wei WF, Zhou CF, Wu XG (2017). MicroRNA-221-3p, a TWIST2 target, promotes cervical cancer metastasis by directly targeting THBS2. Cell Death Dis.

[113] Zhou CF, Ma J, Huang L (2019). Cervical squamous cell carcinoma-secreted exosomal miR-221-3p promotes lymphangiogenesis and lymphatic metastasis by targeting VASH1. Oncogene.

[114] Yang H, Hu H, Gou Y (2018). Combined detection of Twist1, Snail1 and squamous cell carcinoma antigen for the prognostic evaluation of invasion and metastasis in cervical squamous cell carcinoma. Int J Clin Oncol.

[115] Valle-Mendiola A, Gutierrez-Hoya A, Lagunas-Cruz Mdel C (2016). Pleiotropic Effects of IL-2 on Cancer: its role in cervical cancer. Mediators Inflamm.

[116] Zhu H, Luo H, Shen Z (2016). Transforming growth factor-beta1 in carcinogenesis, progression, and therapy in cervical cancer. Tumour Biol.

[117] Ng SC, Wang PH, Lee YC (2019). Impact of matrix metalloproteinase-11 gene polymorphisms on development and clinicopathologcial variables of uterine cervical cancer in Taiwanese women. Int J Med Sci.

[118] Wang Q, Steger A, Mahner S (2019). The formation and therapeutic update of tumor-associated macrophages in cervical cancer. Int J Mol Sci.

[119] Zhao Z, Li J, Li H (2020). Integrative bioinformatics approaches to screen potential prognostic immune-related genes and drugs in the cervical cancer microenvironment. Front Genet.

[120] Oany AR, Mia M, Pervin T (2021). Integrative systems biology approaches to identify potential biomarkers and pathways of cervical cancer. J Pers Med.

[121] Shang C, Zhu W, Liu T (2016). Characterization of long non-coding RNA expression profiles in lymph node metastasis of early-stage cervical cancer. Oncol Rep.

[122] Murata T, Mekada E, Hoffman RM (2017). Reconstitution of a metastatic-resistant tumor microenvironment with cancer-associated fibroblasts enables metastasis. Cell Cycle.

[123] Wei H, Dong C, Shen Z (2020). Kallikrein-related peptidase (KLK10) cessation blunts colorectal cancer cell growth and glucose metabolism by regulating the PI3K/Akt/mTOR pathway. Neoplasma.

[124] Wei WF, Chen XJ, Liang LJ (2021). Periostin+ cancer-associated fibroblasts promote lymph node metastasis by impairing the lymphatic endothelial barriers in cervical squamous cell carcinoma. Mol Oncol.

[125] Zhang R, Lu H, Lyu YY (2017). E6/E7-P53-POU2F1-CTHRC1 axis promotes cervical cancer metastasis and activates Wnt/PCP pathway. Sci Rep.

[126] Kaewprag J, Umnajvijit W, Ngamkham J, Ponglikitmongkol M (2013). HPV16 oncoproteins promote cervical cancer invasiveness by upregulating specific matrix metalloproteinases. PLoS ONE.

[127] Shang C, Wang W, Liao Y (2018). LNMICC promotes nodal metastasis of cervical cancer by reprogramming fatty acid metabolism. Cancer Res.

[128] Rotman J, Koster BD, Jordanova ES, Heeren AM, de Gruijl TD (2019). Unlocking the therapeutic potential of primary tumor-draining lymph nodes. Cancer Immunol Immunother.

[129] Gerner MY, Casey KA, Kastenmuller W, Germain RN (2017). Dendritic cell and antigen dispersal landscapes regulate T cell immunity. J Exp Med.

[130] Noubade R, Majri-Morrison S, Tarbell KV (2019). Beyond cDC1: emerging roles of DC crosstalk in cancer immunity. Front Immunol.

[131] Jones D, Pereira ER, Padera TP (2018). Growth and immune evasion of lymph node metastasis. Front Oncol.

[132] Heeren AM, de Boer E, Bleeker MC (2015). Nodal metastasis in cervical cancer occurs in clearly delineated fields of immune suppression in the pelvic lymph catchment area. Oncotarget.

[133] Hazelbag S, Gorter A, Kenter GG, van den Broek L, Fleuren G (2002). Transforming growth factor-beta1 induces tumor stroma and reduces tumor infiltrate in cervical cancer. Hum Pathol.

[134] Heusinkveld M, de Vos van Steenwijk PJ, Goedemans R (2011). M2 macrophages induced by prostaglandin E2 and IL-6 from cervical carcinoma are switched to activated M1 macrophages by CD4+ Th1 cells. J Immunol.

[135] Kim MH, Seo SS, Song YS (2003). Expression of cyclooxygenase-1 and -2 associated with expression of VEGF in primary cervical cancer and at metastatic lymph nodes. Gynecol Oncol.

[136] Karpanen T, Egeblad M, Karkkainen MJ (2001). Vascular endothelial growth factor C promotes tumor lymphangiogenesis and intralymphatic tumor growth. Cancer Res.

[137] Heeren AM, Kenter GG, Jordanova ES, de Gruijl TD (2015). CD14(+) macrophage-like cells as the linchpin of cervical cancer perpetrated immune suppression and early metastatic spread: a new therapeutic lead?. Oncoimmunology.

[138] Zijlmans HJ, Fleuren GJ, Baelde HJ, Eilers PH, Kenter GG, Gorter A (2006). The absence of CCL2 expression in cervical carcinoma is associated with increased survival and loss of heterozygosity at 17q11.2. J Pathol.

[139] Heeren AM, Koster BD, Samuels S (2015). High and interrelated rates of PD-L1+CD14+ antigen-presenting cells and regulatory T cells mark the microenvironment of metastatic lymph nodes from patients with cervical cancer. Cancer Immunol Res.

[140] Van de Ven R, Lindenberg JJ, Oosterhoff D, de Gruijl TD (2013). Dendritic Cell Plasticity in Tumor-Conditioned Skin: CD14(+) Cells at the Cross-Roads of Immune Activation and Suppression. Front Immunol.

[141] Lindenberg JJ, Oosterhoff D, Sombroek CC (2013). IL-10 conditioning of human skin affects the distribution of migratory dendritic cell subsets and functional T cell differentiation. PLoS ONE.

[142] Heeren AM, Rotman J, Stam AGM (2019). Efficacy of PD-1 blockade in cervical cancer is related to a CD8(+)FoxP3(+)CD25(+) T-cell subset with operational effector functions despite high immune checkpoint levels. J Immunother Cancer.

[143] Van Pul KM, Fransen MF, van de Ven R, de Gruijl TD (2021). Immunotherapy goes local: the central role of lymph nodes in driving tumor infiltration and efficacy. Front Immunol.

[144] Alonso R, Flament H, Lemoine S (2018). Induction of anergic or regulatory tumor-specific CD4(+) T cells in the tumor-draining lymph node. Nat Commun.

[145] Yang P, Ruan Y, Yan Z, Gao Y, Yang H, Wang S (2021). Comprehensive analysis of lymph nodes metastasis associated genes in cervical cancer and its significance in treatment and prognosis. BMC Cancer.

[146] Jin J, Zhang Z, Zhang S (2018). Fatty acid binding protein 4 promotes epithelial-mesenchymal transition in cervical squamous cell carcinoma through AKT/GSK3beta/Snail signaling pathway. Mol Cell Endocrinol.

[147] Zhang W, Wu Q, Wang C, Yang L, Liu P, Ma C (2018). AKIP1 promotes angiogenesis and tumor growth by upregulating CXC-chemokines in cervical cancer cells. Mol Cell Biochem.

[148] Wang S, Li J, Xie J (2018). Programmed death ligand 1 promotes lymph node metastasis and glucose metabolism in cervical cancer by activating integrin beta4/SNAI1/SIRT3 signaling pathway. Oncogene.

[149] Haile ST, Bosch JJ, Agu NI (2011). Tumor cell programmed death ligand 1-mediated T cell suppression is overcome by coexpression of CD80. J Immunol.

[150] Chaudhari S, Dey Pereira S, Asare-Warehene M (2021). Comorbidities and inflammation associated with ovarian cancer and its influence on SARS-CoV-2 infection. J Ovarian Res.

[151] Gardner AB, Charo LM, Mann AK, Kapp DS, Eskander RN, Chan JK (2020). Ovarian, uterine, and cervical cancer patients with distant metastases at diagnosis: most common locations and outcomes. Clin Exp Metastasis.

[152] Chen X, Chen L, Zhu H, Tao J (2020). Risk factors and prognostic predictors for Cervical Cancer patients with lung metastasis. J Cancer.

[153] Ki EY, Lee KH, Park JS, Hur SY (2016). A clinicopathological review of pulmonary metastasis from uterine cervical cancer. Cancer Res Treat.

[154] Wang CW, Wu TI, Yu CT (2009). Usefulness of p16 for differentiating primary pulmonary squamous cell carcinoma from cervical squamous cell carcinoma metastatic to the lung. Am J Clin Pathol.

[155] Massague J, Obenauf AC (2016). Metastatic colonization by circulating tumour cells. Nature.

[156] Joyce JA, Pollard JW (2009). Microenvironmental regulation of metastasis. Nat Rev Cancer.

[157] Takebe N, Harris PJ, Warren RQ, Ivy SP (2011). Targeting cancer stem cells by inhibiting Wnt, Notch, and Hedgehog pathways. Nat Rev Clin Oncol.

[158] Lintz M, Munoz A, Reinhart-King CA (2017). The mechanics of single cell and collective migration of tumor cells. J Biomech Eng.

[159] Pein M, Insua-Rodriguez J, Hongu T (2020). Metastasis-initiating cells induce and exploit a fibroblast niche to fuel malignant colonization of the lungs. Nat Commun.

[160] Hsu YL, Yen MC, Chang WA (2019). CXCL17-derived CD11b(+)Gr-1(+) myeloid-derived suppressor cells contribute to lung metastasis of breast cancer through platelet-derived growth factor-BB. Breast Cancer Res.

[161] Giannou AD, Marazioti A, Kanellakis NI (2017). NRAS destines tumor cells to the lungs. EMBO Mol Med.

[162] Matsuyama T, Tsukamoto N, Imachi M, Nakano H (1989). Bone metastasis from cervix cancer. Gynecol Oncol.

[163] Thanapprapasr D, Nartthanarung A, Likittanasombut P (2010). Bone metastasis in cervical cancer patients over a 10-year period. Int J Gynecol Cancer.

[164] Dewdney A, Selvarajah U (2010). A 'hot' leg: a rare case of isolated long bone metastases from cervical cancer. Anticancer Res.

[165] Kolb AD, Bussard KM (2019). The bone extracellular matrix as an ideal milieu for cancer cell metastases. Cancers (Basel).

[166] Somaiah C, Kumar A, Mawrie D (2015). Collagen Promotes Higher Adhesion, Survival and Proliferation of Mesenchymal Stem Cells. PLoS ONE.

[167] Sekita A, Matsugaki A, Nakano T (2017). Disruption of collagen/apatite alignment impairs bone mechanical function in osteoblastic metastasis induced by prostate cancer. Bone.

[168] Romero-Moreno R, Curtis KJ, Coughlin TR (2019). The CXCL5/CXCR2 axis is sufficient to promote breast cancer colonization during bone metastasis. Nat Commun.

[169] Yin JJ, Mohammad KS, Kakonen SM (2003). A causal role for endothelin-1 in the pathogenesis of osteoblastic bone metastases. Proc Natl Acad Sci U S A.

[170] Fagundes H, Perez CA, Grigsby PW, Lockett MA (1992). Distant metastases after irradiation alone in carcinoma of the uterine cervix. Int J Radiat Oncol Biol Phys.

[171] Tosoni A, Ermani M, Brandes AA (2004). The pathogenesis and treatment of brain metastases: a comprehensive review. Crit Rev Oncol Hematol.

[172] Divine LM, Kizer NT, Hagemann AR (2016). Clinicopathologic characteristics and survival of patients with gynecologic malignancies metastatic to the brain. Gynecol Oncol.

[173] Lambert AW, Pattabiraman DR, Weinberg RA (2017). Emerging Biological Principles of Metastasis. Cell.

[174] Kim GE, Lee SW, Suh CO (1998). Hepatic metastases from carcinoma of the uterine cervix. Gynecol Oncol.

[175] Li H, Pang Y, Cheng X (2020). Surgery of primary sites for stage IVB cervical cancer patients receiving chemoradiotherapy: a population-based study. J Gynecol Oncol.

[176] Ramachandran D, Dork T (2021). Genomic risk factors for cervical cancer. Cancers (Basel).

[177] Nissar S, Sameer AS, Banday MZ, Sameer (2021). Genetic Polymorphisms of Essential Immune Pathogenic Response Genes and Risk of Cervical Cancer. Genetic Polymorphism and cancer susceptibility.

[178] Chen D, Gyllensten U (2015). Lessons and implications from association studies and post-GWAS analyses of cervical cancer. Trends Genet.

[179] Chen X, Jiang J, Shen H, Hu Z (2011). Genetic susceptibility of cervical cancer. J Biomed Res.

[180] Lin W, Feng M, Li X (2017). Transcriptome profiling of cancer and normal tissues from cervical squamous cancer patients by deep sequencing. Mol Med Rep.

[181] Wu X, Peng L, Zhang Y (2019). Identification of key genes and pathways in cervical cancer by bioinformatics analysis. Int J Med Sci.

[182] Pardini B, De Maria D, Francavilla A, Di Gaetano C, Ronco G, Naccarati A (2018). MicroRNAs as markers of progression in cervical cancer: a systematic review. BMC Cancer.

[183] Molina MA, Carosi Diatricch L, Castany Quintana M, Melchers WJ, Andralojc KM (2020). Cervical cancer risk profiling: molecular biomarkers predicting the outcome of hrHPV infection. Expert Rev Mol Diagn.

[184] Gomez-Gomez Y, Organista-Nava J, Gariglio P (2013). Deregulation of the miRNAs expression in cervical cancer: human papillomavirus implications. Biomed Res Int.

[185] Banno K, Iida M, Yanokura M (2014). MicroRNA in cervical cancer: OncomiRs and tumor suppressor miRs in diagnosis and treatment. ScientificWorldJournal.

[186] Laengsri V, Kerdpin U, Plabplueng C, Treeratanapiboon L, Nuchnoi P (2018). Cervical cancer markers: epigenetics and microRNAs. Lab Med.

[187] Wang JY, Chen LJ (2019). Biosci Rep.

[188] Kim HJ, Lee DW, Yim GW (2015). Long non-coding RNA HOTAIR is associated with human cervical cancer progression. Int J Oncol.

[189] Zhong Q, Lu M, Yuan W (2021). Eight-lncRNA signature of cervical cancer were identified by integrating DNA methylation, copy number variation and transcriptome data. J Transl Med.

[190] Haddada M, Draoui H, Deschamps L (2018). Kallikrein-related peptidase 7 overexpression in melanoma cells modulates cell adhesion leading to a malignant phenotype. Biol Chem.

[191] Sharma S, Munger K (2018). Expression of the cervical carcinoma expressed PCNA regulatory (CCEPR) long noncoding RNA is driven by the human papillomavirus E6 protein and modulates cell proliferation independent of PCNA. Virology.

[192] Liu S, Song L, Zeng S, Zhang L (2016). MALAT1-miR-124-RBG2 axis is involved in growth and invasion of HR-HPV-positive cervical cancer cells. Tumour Biol.

[193] He H, Liu X, Liu Y (2019). Human papillomavirus E6/E7 and long noncoding RNA TMPOP2 mutually upregulated gene expression in cervical cancer cells. J Virol.

[194] Hong H, Zhu H, Zhao S (2019). The novel circCLK3/miR-320a/FoxM1 axis promotes cervical cancer progression. Cell Death Dis.

[195] Khandelwal A, Sharma U, Barwal TS (2021). Circulating miR-320a Acts as a Tumor Suppressor and Prognostic Factor in Non-small Cell Lung Cancer. Front Oncol.

[196] Ji F, Du R, Chen T (2020). Circular RNA circSLC26A4 Accelerates Cervical Cancer Progression via miR-1287-5p/HOXA7 Axis. Mol Ther Nucleic Acids.

[197] Fan S, Zhao S, Gao X (2020). Circular RNA circGSE1 Promotes Cervical Cancer Progression Through miR-138-5p/Vimentin. Onco Targets Ther.

[198] Li X, Ma N, Zhang Y (2020). Circular RNA circNRIP1 promotes migration and invasion in cervical cancer by sponging miR-629-3p and regulating the PTP4A1/ERK1/2 pathway. Cell Death Dis.

[199] Ma X, Wang C, Chen J, Wei D, Yu F, Sun J (2021). circAGFG1 sponges miR-28-5p to promote non-small-cell lung cancer progression through modulating HIF-1alpha level. Open Med (Wars).

[200] Ma ML, Zhang HY, Zhang SY, Yi XL (2021). LncRNA CDKN2BAS1 sponges miR-28-5p to regulate proliferation and inhibit apoptosis in colorectal cancer. Oncol Rep.

[201] Mole S, McFarlane M, Chuen-Im T, Milligan SG, Millan D, Graham SV (2009). RNA splicing factors regulated by HPV16 during cervical tumour progression. J Pathol.

[202] Santin AD, Zhan F, Bignotti E (2005). Gene expression profiles of primary HPV16- and HPV18-infected early stage cervical cancers and normal cervical epithelium: identification of novel candidate molecular markers for cervical cancer diagnosis and therapy. Virology.

[203] Zhao M, Huang W, Zou S, Shen Q, Zhu X (2020). A five-genes-based prognostic signature for cervical cancer overall survival prediction. Int J Genomics.

[204] Ge Y, Zhang C, Xiao S (2018). Identification of differentially expressed genes in cervical cancer by bioinformatics analysis. Oncol Lett.

[205] Chen D, Juko-Pecirep I, Hammer J (2013). Genome-wide association study of susceptibility loci for cervical cancer. J Natl Cancer Inst.

[206] Gu P, Goodwin B, Chung AC (2005). Orphan nuclear receptor LRH-1 is required to maintain Oct4 expression at the epiblast stage of embryonic development. Mol Cell Biol.

[207] Heng JC, Feng B, Han J (2010). The nuclear receptor Nr5a2 can replace Oct4 in the reprogramming of murine somatic cells to pluripotent cells. Cell Stem Cell.

[208] Cooke SL, Temple J, Macarthur (2011). Intra-tumour genetic heterogeneity and poor chemoradiotherapy response in cervical cancer. Br J Cancer.

[209] Ortiz-Sanchez E, Santiago-Lopez L, Cruz-Dominguez VB (2016). Characterization of cervical cancer stem cell-like cells: phenotyping, stemness, and human papilloma virus co-receptor expression. Oncotarget.

[210] Ayob AZ, Ramasamy TS (2018). Cancer stem cells as key drivers of tumour progression. J Biomed Sci.

[211] Thiery JP (2002). Epithelial-mesenchymal transitions in tumour progression. Nat Rev Cancer.

[212] Pastushenko I, Blanpain C (2019). EMT transition states during tumor progression and metastasis. Trends Cell Biol.

[213] De Craene B, Berx G (2013). Regulatory networks defining EMT during cancer initiation and progression. Nat Rev Cancer.

[214] Tian R, Li X, Gao Y, Li Y, Yang P, Wang K (2018). Identification and validation of the role of matrix metalloproteinase-1 in cervical cancer. Int J Oncol.

[215] Edgar R, Domrachev M, Lash AE (2002). Gene expression omnibus: NCBI gene expression and hybridization array data repository. Nucleic Acids Res.

[216] Ritchie ME, Phipson B, Wu D (2015). limma powers differential expression analyses for RNA-sequencing and microarray studies. Nucleic Acids Res.

[217] Conway JR, Lex A, Gehlenborg N (2017). UpSetR: an R package for the visualization of intersecting sets and their properties. Bioinformatics.

[218] Szklarczyk D, Franceschini A, Wyder S (2015). STRING v10: protein-protein interaction networks, integrated over the tree of life. Nucleic Acids Res.

[219] Chin CH, Chen SH, Wu HH, Ho CW, Ko MT, Lin CY (2014). cytoHubba: identifying hub objects and sub-networks from complex interactome. BMC Syst Biol.

[220] Raudvere U, Kolberg L, Kuzmin I (2019). g:Profiler: a web server for functional enrichment analysis and conversions of gene lists (2019 update). Nucleic Acids Res.

[221] Chen EY, Tan CM, Kou Y (2013). Enrichr: interactive and collaborative HTML5 gene list enrichment analysis tool. BMC Bioinformatics.

[222] Zhou G, Soufan O, Ewald J, Hancock REW, Basu N, Xia J (2019). NetworkAnalyst 3.0: a visual analytics platform for comprehensive gene expression profiling and meta-analysis. Nucleic Acids Res.

[223] Andrighetti T, Bohar B, Lemke N, Sudhakar P, Korcsmaros T (2020). MicrobioLink: an integrated computational pipeline to infer functional effects of microbiome-host interactions. Cells.

[224] Mayakonda A, Lin DC, Assenov Y, Plass C, Koeffler HP (2018). Maftools: efficient and comprehensive analysis of somatic variants in cancer. Genome Res.

[225] Uhlen M, Oksvold P, Fagerberg L (2010). Towards a knowledge-based Human Protein Atlas. Nat Biotechnol.

[226] Dean CJ, Bockmann MR, Hopwood JJ, Brooks DA, Meikle PJ (2006). Detection of mucopolysaccharidosistype II by measurement of iduronate-2-sulfatase in dried blood spots and plasma samples. Clin Chem.

[227] Singh V, Jha KK, M JK, Kumar RV, Raghunathan V, Bhat R (2019). Iduronate-2-Sulfatase-regulated dermatan sulfate levels potentiate the invasion of breast cancer epithelia through collagen matrix. J Clin Med.

[228] Gao X, Gao CX, Liu GX, Hu J (2016). MAP4K4: an emerging therapeutic target in cancer. Cell Biosci.

[229] Chuang HC, Wang X, Tan TH (2016). MAP4K family kinases in immunity and inflammation. Adv Immunol.

[230] Danai LV, Flach RJ, Virbasius JV (2015). Inducible deletion of protein kinase Map4k4 in obese mice improves insulin sensitivity in liver and adipose tissues. Mol Cell Biol.

[231] Virbasius JV, Czech MP (2016). Map4k4 signaling nodes in metabolic and cardiovascular diseases. Trends Endocrinol Metab.

[232] Xue Y, Wang X, Li Z, Gotoh N, Chapman D, Skolnik EY (2001). Mesodermal patterning defect in mice lacking the Ste20 NCK interacting kinase (NIK). Development.

[233] Feng XJ, Pan Q, Wang SM (2016). MAP4K4 promotes epithelial-mesenchymal transition and metastasis in hepatocellular carcinoma. Tumour Biol.

[234] Huang H, Han Q, Zheng H (2021). MAP4K4 mediates the SOX6-induced autophagy and reduces the chemosensitivity of cervical cancer. Cell Death Dis.

[235] Hao JM, Chen JZ, Sui HM (2010). A five-gene signature as a potential predictor of metastasis and survival in colorectal cancer. J Pathol.

[236] Mei J, Wang DH, Wang LL, Chen Q, Pan LL, Xia L (2018). MicroRNA-200c suppressed cervical cancer cell metastasis and growth via targeting MAP4K4. Eur Rev Med Pharmacol Sci.

[237] Zhao G, Wang B, Liu Y (2013). miRNA-141, downregulated in pancreatic cancer, inhibits cell proliferation and invasion by directly targeting MAP4K4. Mol Cancer Ther.

[238] Liu YF, Qu GQ, Lu YM (2016). Silencing of MAP4K4 by short hairpin RNA suppresses proliferation, induces G1 cell cycle arrest and induces apoptosis in gastric cancer cells. Mol Med Rep.

[239] Liu AW, Cai J, Zhao XL (2011). ShRNA-Targeted MAP4K4 Inhibits Hepatocellular Carcinoma Growth. Clin Cancer Res.

[240] Yang N, Wang Y, Hui L, Li X, Jiang X (2015). Silencing SOX2 Expression by RNA Interference Inhibits Proliferation, Invasion and Metastasis, and Induces Apoptosis through MAP4K4/JNK Signaling Pathway in Human Laryngeal Cancer TU212 Cells. J Histochem Cytochem.

[241] Collins CS, Hong JY, Sapinoso L (2006). A small interfering RNA screen for modulators of tumor cell motility identifies MAP4K4 as a promigratory kinase. Proc Natl Acad Sci USA.

[242] Wang T, Jin H, Hu J (2020). COL4A1 promotes the growth and metastasis of hepatocellular carcinoma cells by activating FAK-Src signaling. J Exp Clin Cancer Res.

[243] JingSong H, Hong G, Yang J (2017). siRNA-mediated suppression of collagen type iv alpha 2 (COL4A2) mRNA inhibits triple-negative breast cancer cell proliferation and migration. Oncotarget.

[244] Li Z, Yu CP, Zhong Y (2012). Sam68 expression and cytoplasmic localization is correlated with lymph node metastasis as well as prognosis in patients with early-stage cervical cancer. Ann Oncol.

[245] Barlat I, Maurier F, Duchesne M, Guitard E, Tocque B, Schweighoffer F (1997). A role for Sam68 in cell cycle progression antagonized by a spliced variant within the KH domain. J Biol Chem.

[246] Lukong KE, Larocque D, Tyner AL, Richard S (2005). Tyrosine phosphorylation of sam68 by breast tumor kinase regulates intranuclear localization and cell cycle progression. J Biol Chem.

[247] Fu K, Sun X, Xia X (2019). Sam68 is required for the growth and survival of nonmelanoma skin cancer. Cancer Med.

[248] Chen S, Li J, Zhou P, Zhi X (2020). SPTBN1 and cancer, which links?. J Cell Physiol.

[249] Chen M, Zeng J, Chen S (2020). SPTBN1 suppresses the progression of epithelial ovarian cancer via SOCS3-mediated blockade of the JAK/STAT3 signaling pathway. Aging (Albany NY).

[250] Yang S, Sun Y, Jiang D (2021). MiR-362 suppresses cervical cancer progression via directly targeting BAP31 and activating TGFbeta/Smad pathway. Cancer Med.

[251] Chen S, Liu C (2021). SPTBN1 inhibits growth and epithelial-mesenchymal transition in breast cancer by downregulating miR-21. Eur J Pharmacol.

[252] Zhi X, Lin L, Yang S (2015). betaII-Spectrin (SPTBN1) suppresses progression of hepatocellular carcinoma and Wnt signaling by regulation of Wnt inhibitor kallistatin. Hepatology.

[253] Yousef GM, Scorilas A, Magklara A, Soosaipillai A, Diamandis EP (2000). The KLK7 (PRSS6) gene, encoding for the stratum corneum chymotryptic enzyme is a new member of the human kallikrein gene family - genomic characterization, mapping, tissue expression and hormonal regulation. Gene.

[254] Cane S, Bignotti E, Bellone S (2004). The novel serine protease tumor-associated differentially expressed gene-14 (KLK8/Neuropsin/Ovasin) is highly overexpressed in cervical cancer. Am J Obstet Gynecol.

[255] Jin H, Nagai N, Shigemasa K (2006). Expression of tumor-associated differentially expressed Gene-14 (TADG-14/KLK8) and its protein hK8 in uterine endometria and endometrial carcinomas. Tumour Biol.

[256] Hua Q, Sun Z, Liu Y (2021). KLK8 promotes the proliferation and metastasis of colorectal cancer via the activation of EMT associated with PAR1. Cell Death Dis.

[257] Liu CJ, Liu TY, Kuo LT (2008). Differential gene expression signature between primary and metastatic head and neck squamous cell carcinoma. J Pathol.

[258] Michaelidou K, Ardavanis A, Scorilas A (2015). Clinical relevance of the deregulated kallikrein-related peptidase 8 mRNA expression in breast cancer: a novel independent indicator of disease-free survival. Breast Cancer Res Treat.

[259] Rajapakse S, Ogiwara K, Takano N, Moriyama A, Takahashi T (2005). Biochemical characterization of human kallikrein 8 and its possible involvement in the degradation of extracellular matrix proteins. FEBS Lett.

[260] Li W, Zhao Y, Ren L, Wu X (2014). Serum human kallikrein 7 represents a new marker for cervical cancer. Med Oncol.

[261] Santin AD, Cane S, Bellone S (2004). The serine protease stratum corneum chymotryptic enzyme (kallikrein 7) is highly overexpressed in squamous cervical cancer cells. Gynecol Oncol.

[262] Raju I, Kaushal GP, Haun RS (2016). Epigenetic regulation of KLK7 gene expression in pancreatic and cervical cancer cells. Biol Chem.

[263] Xiang F, Wang Y, Cao C (2022). The role of kallikrein 7 in tumorigenesis. Curr Med Chem.

[264] Ramani VC, Haun RS (2008). Expression of kallikrein 7 diminishes pancreatic cancer cell adhesion to vitronectin and enhances urokinase-type plasminogen activator receptor shedding. Pancreas.

[265] Kumar DV, Sivaranjani Y, Rao GV (2020). Immunohistochemical expression of kallikrein 7 in oral squamous cell carcinoma. J Oral Maxillofac Pathol.

[266] Prezas P, Scorilas A, Yfanti C (2006). The role of human tissue kallikreins 7 and 8 in intracranial malignancies. Biol Chem.

[267] Yousef GM, Obiezu CV, Luo LY (2005). Human tissue kallikreins: from gene structure to function and clinical applications. Adv Clin Chem.

[268] Goyal J, Smith KM, Cowan JM, Wazer DE, Lee SW, Band V (1998). The role for NES1 serine protease as a novel tumor suppressor. Cancer Res.

[269] Sidiropoulos M, Pampalakis G, Sotiropoulou G, Katsaros D, Diamandis EP (2005). Downregulation of human kallikrein 10 (KLK10/NES1) by CpG island hypermethylation in breast, ovarian and prostate cancers. Tumour Biol.

[270] Roman-Gomez J, Jimenez-Velasco A, Agirre X (2004). The normal epithelial cell-specific 1 (NES1) gene, a candidate tumor suppressor gene on chromosome 19q13.3-4, is downregulated by hypermethylation in acute lymphoblastic leukemia. Leukemia.

[271] Zheng H, Zhang W, Wang X, Zhao G (2012). Enhancement of kallikrein-related peptidase 10 expression attenuates proliferation and invasiveness of human tongue cancer cells in vitro. Nan Fang Yi Ke Da Xue Xue Bao.

[272] Lin CL, Ying TH, Yang SF (2020). Transcriptional suppression of miR-7 by MTA2 induces Sp1-mediated KLK10 expression and metastasis of cervical cancer. Mol Ther Nucleic Acids.

[273] Sano K, Tanihara H, Heimark RL (1993). Protocadherins: a large family of cadherin-related molecules in central nervous system. EMBO J.

[274] Cao J, Wang M, Wang T (2017). CCAAT enhancer binding protein beta has a crucial role in regulating breast cancer cell growth via activating the TGF-beta-Smad3 signaling pathway. Exp Ther Med.

[275] Zhou X, Updegraff BL, Guo Y (2017). PROTOCADHERIN 7 Acts through SET and PP2A to Potentiate MAPK Signaling by EGFR and KRAS during Lung Tumorigenesis. Cancer Res.

[276] Shishodia G, Koul S, Koul HK (2019). Protocadherin 7 is overexpressed in castration resistant prostate cancer and promotes aberrant MEK and AKT signaling. Prostate.

[277] Chen HF, Ma RR, He JY (2017). Protocadherin 7 inhibits cell migration and invasion through E-cadherin in gastric cancer. Tumour Biol.

[278] Zhang S, Fu X (2021). The clinical significance and biological function of PCDH7 in cervical cancer. Cancer Manag Res.

[279] Li Q, Li B, Lu CL, Wang JY, Gao M, Gao W (2021). LncRNA LINC01857 promotes cell growth and diminishes apoptosis via PI3K/mTOR pathway and EMT process by regulating miR-141-3p/MAP4K4 axis in diffuse large B-cell lymphoma. Cancer Gene Ther.

[280] Fu Y, Liu X, Chen Q (2018). Downregulated miR-98-5p promotes PDAC proliferation and metastasis by reversely regulating MAP4K4. J Exp Clin Cancer Res.

[281] Kang SD, Chatterjee S, Alam S (2018). Effect of productive human papillomavirus 16 infection on global gene expression in cervical epithelium. J Virol.

[282] Delaunay T, Deschamps L, Haddada M (2017). Aberrant expression of kallikrein-related peptidase 7 is correlated with human melanoma aggressiveness by stimulating cell migration and invasion. Mol Oncol.

[283] Mo L, Zhang J, Shi J (2010). Human kallikrein 7 induces epithelial-mesenchymal transition-like changes in prostate carcinoma cells: a role in prostate cancer invasion and progression. Anticancer Res.

[284] Xu LJ, Duan Y, Wang P, Yin HQ (2018). MiR-199b-5p promotes tumor growth and metastasis in cervical cancer by down-regulating KLK10. Biochem Biophys Res Commun.

[285] Hu J, Lei H, Fei X (2015). NES1/KLK10 gene represses proliferation, enhances apoptosis and down-regulates glucose metabolism of PC3 prostate cancer cells. Sci Rep.

[286] Chen X, Zhang L, Yuan M (2020). Sam68 promotes the progression of human breast cancer through inducing activation of EphA3. Curr Cancer Drug Targets.

